# Adaptogenic and Immunomodulatory Activity of Ashwagandha Root Extract: An Experimental Study in an Equine Model

**DOI:** 10.3389/fvets.2020.541112

**Published:** 2020-09-29

**Authors:** G. Priyanka, B. Anil Kumar, M. Lakshman, V. Manvitha, B. Kala Kumar

**Affiliations:** ^1^Department of Veterinary Pharmacology and Toxicology, College of Veterinary Science Rajendranagar, Hyderabad, India; ^2^Department of Veterinary Pathology, College of Veterinary Science, Hyderabad, India

**Keywords:** horses, stress, Ashwagandha root extract, adaptogen, cortisol, IL-6 3, Ashwagandha (*Withania somnifera*)

## Abstract

Ashwagandha (*Withania somnifera* L. Dunal.) is an important “*Rasayana*” of Ayurveda. The roots are extensively used as an adaptogen and for different health issues. Anti-inflammatory, antioxidant, and immune-stimulating effects of Ashwagandha are well-documented. The present study aimed to evaluate the clinical efficacy of Ashwagandha root extract as an adaptogen against various types of stress in horses. A total of 24 Kathiawari horses were selected and randomly divided into four groups. All the horses were provided with normal feed and water *ad libitum*. Group 1 (G1) was treated as the control group, and the horses were given a normal diet. Group 2 (G2), Group 3 (G3), and Group 4 (G4) horses received varying doses of Ashwagandha root extract along with the normal diet. All the animals were subjected to different types of stress including exercise-induced stress, separation, and noise stress on three different days and evaluated for various hematological, biochemical, hormonal, and immunological parameters. Over the 21 days, a statistically significant (*p* < 0.05) increase in total erythrocyte count, total leucocyte count, hemoglobin content, lymphocyte percentage, reduced glutathione, and superoxide dismutase activities was observed. A statistically significant (*p* < 0.05) decrease in cortisol, epinephrine, glucose, triglycerides, creatinine, IL-6, alanine aminotransferase, and aspartate aminotransferase was observed in the Ashwagandha treated groups (G2, G3, and G4) when compared to the control group (G1). The results suggest that Ashwagandha root extract has potent hemopoietic, antioxidant, adaptogenic, and immune-stimulant properties.

## Introduction

Management of various environmental stress remains a great challenge for animals. Altering environmental stimulations affects the homeostasis and impact the hormonal secretion and functions. Acute or chronic stimulations can change the physiological conditions with time. Stress is a non-specific response of humans and animals that may appear due to unpleasant stimuli or perception (1). Mostly, stress refers to the unacceptance or non-adaptation of a particular negative stimulation and expressed through acute or chronic behavioral changes. However, the degree of behavior change differs from species to species and individual to individual. Negative emotion induces stress levels in both humans and animals (2, 3).

Evolutionarily, horses are prey animals, maintain a herd, follow social facilitation, and are adapted to have a fight-or-flight response. Thus, they are emotional, highly sensitive, and attentive and responsive toward the environmental stimulations. In equine species, elevated stress is observed in the presence of other species, including humans. An unpredictable and uncontrollable environmental condition could initiate stress and can be responsible for non-specific functional and behavior changes (4). However, a specific stress response is dependent on the interpretation of a particular situation, rather than the actual situation itself (5). Several rapid physiological responses including endocrine, respiratory, cardiovascular occur through the activation of the sympathetic nervous system due to stress (6).

Often, due to stress, horses may display “stable vice” by expressing fear, anxiety due to social separation, pain, transportation, and other issues (7). Horses are highly responsive to exercise-induced stress, noise, and social isolation. Stress augments various physiological responses such as altered heart rate, temperature, breathing rate, hormonal surge, and behavioral response including fleeing, kicking and biting, stall walking, shaking, spooking, and bolting (8). Chronic stress in horses can be reflected through indigestion and gastric ulcer formation, altered or stereotype behavior, immune suppression, prone to infections, and weight loss (9).

Therefore, assessment of the stress conditions and behavior of horses is important to improve their physical and psychological fitness. Analysis of the stress hormones is required to understand the level of stress and relevant physiological effect. Under stress, an increase in cortisol, adrenaline, nor-adrenaline, and reduction in serotonin activity was reported in horses (10). An upsurge in the cortisol hormone in horses was also reported due to exercise-induced stress (11).

Physical exercise and stress induce biochemical adaptations and alter blood constituents. A report suggests that exercise in horses alters the packed cell volume, hemoglobin, total erythrocyte, and leukocyte count, total proteins, cortisol, alanine aminotransferase activity, and aspartate aminotransferase activity (12). As a result of oxidative stress, a proportionate increment in the reactive oxygen species (ROS) production during exercise is reported. During transportation, increased oxidative stress markers Malondialdehyde and superoxide dismutase, increased heart rate, and respiratory rate are reported in horses (13). Prolonged exercise resulted in an upsurge of tumor necrosis factor-α (TNF-α), and interleukin-6 (IL-6). In trained horses, after acute exercise, increment in IL-6 was reported (14).

Hence, supplements having benefits in stress management can maintain physical and psychological balance in horses. Herbal supplements are natural and are recognized by leading health organizations (15). Ashwagandha (*Withania somnifera* L. Dunal) could be a good supplement for being natural and adaptogen and acting as a revitalizer and having a soothing or somniferous effect. Ashwagandha is a perennial herb of the tropical region and is traditionally respected as a “*Rasayana”* in Ayurveda (16). Withania root extract is known to promote health through revitalization, disease resistance, and improved mental well-being (16). Polyherbal supplement that included Ashwagandha was reported as a reliable antioxidant source in horses (17). The present study was designed to determine the antioxidant, hematological, and immunomodulatory effect of Ashwagandha root extract in varying doses on horses exposed to various kinds of stress.

## Materials and Methods

### Study Animals

During the 21-days study, twenty-four (24) healthy Kathiawari horses of either sex, aged between 5 and 10 years, were selected and housed at the Equine farm of Hyderabad, India. All the horses were fed with a normal diet consisting of concentrates (6.0–8.0 kg/days), green grass (6.0–7.0 kg/days), and hay (1.5–2.0 kg/days), thrice daily, and water was provided *ad libitum* throughout the study.

### Ethical Consideration

The study was approved by the Institutional Animal Ethics Committee (IAEC) at the College of Veterinary Science, P.V.N.R. Telangana State Veterinary University, Hyderabad (I/2018-38A/IAEC/CVSc, Hyderabad, dated: 16/07/2018) and Committee for Control and Supervision of Experiments on Animals (CPCSEA), New Delhi, India. The animals were under regular observation by the experienced veterinarian.

### Investigational Product

The investigational product used in the study was high-concentration full-spectrum Ashwagandha root powder (KSM-66 Ashwagandha powder), provided by Ixoreal Biomed, CA, USA. The product contains ≥5% of with anolides and is produced by following the Current Good Manufacturing Practices (cGMP) regulations defined by the WHO and US-FDA. All extractions are done following a green chemistry based process that is devoid of any alcohol based extraction. The yellowish powder was approved after the assessment for carcinogenic, mutagenic, teratogenic, and developmental toxicity effect. Each batch is subjected for proper quality check that includes quality assessment in the farm, raw material quality evaluation, Aflatoxin testing, heavy metal analysis, pesticide analysis, bioactive analysis, organoleptic testing, moisture content analysis, microscopic analysis, microbiological testing, pH testing, and ash testing.

### Experimental Design

The horses were divided into 4 groups having 6 animals in each group. The treatment groups 2 (G2), 3(G3), and 4(G4) were given Ashwagandha root extract mixed with jaggery for 21 days at varying doses along with a normal diet. Group 1(G1) was used as a control group and was provided with a normal diet. The animals were given only a normal diet. All the other 3 groups (G2, G3, and G4) were the experimental groups and were given a varying dose of Ashwagandha (2.5 gm/animal, 5 gm/animal, and 10 gm/animal) with jiggery, respectively, for 21 days. After 14 days of Ashwagandha root extract feeding, the horses were subjected to different kinds of stress.

The horses were fed with the testing product (WS) and jaggery on the 15th day. The similar feeding of the horses was continued till the 21st day.

### Stress Induction

#### Exercise-Induced Stress

Initially, the horses were subjected to exercise for 1 h every day. On day 15, the horses were allowed to exercise for 2 h to induce stress. The blood samples were collected within 10 min of stopping the exercise, and stored at −80°C for further analysis.

#### Separation Induced Stress

The horses were subjected to separation stress by isolating them from their regular herd or companion and keeping them in isolated rooms. The animal was isolated for 1 h. The blood samples were collected within 10 min and stored at −80°C for further analysis.

#### Noise-Induced Stress

The horses were subjected to noise stress by playing air horn above the normal intensity. The air horn sound was played through the speakers and the sound levels were above 100 decibels. The sound was played for 1 min by keeping the speakers close to the animal's ears. The reaction of the animal in the form of frightening or disturbing movements was an indication of the endpoint, after which the blood was collected within a minute and stored at −80°C for further analysis.

#### Blood Sample Collection

Blood collection was carried out on Day 0, Day 15, Day 18, and Day 21 in heparinized vials from the horses for each group to analyze all the hematological and other parameters. Blood was collected from the left side of the jugular vein unilaterally after proper restraining and sterilizing the area with surgical spirit. The blood was transferred into two separate vacuum tubes for hematology and serum separation and was stored at −80°C before the analysis. Disposable needles (size: 18) were used for blood collection to maintain sterility and prevent pain-induced stress and hemolysis. The preferable time of blood collection was performed in the early morning before feeding to the horses. Before blood collection, all the horses were kept in the Trevis to avoid stress and anger while collecting blood. The time of feeding was 7–8 a.m., and the collection of blood was done at 11 AM. Serum samples were separated from the blood by using centrifugation at 4,000 rpm for 15 min. The estimation of serum biochemical parameters was followed thereafter.

## Assessments

### Hematological Assays

Total erythrocyte count (TEC), total leucocyte count (TLC), hemoglobin (Hb), packed cell volume (PCV), and lymphocyte percentage were assessed from the blood samples of the horses using hematology analyzer (DH36 VET, Dymind Biotech). Total erythrocyte count and total leucocyte count were expressed in cells/μL, and hemoglobin was expressed in g/dL. Packed cell volume and lymphocyte percentage were presented as percentage (%).

### Hormonal Assay

Serum cortisol, serotonin, and epinephrine were assessed using Enzyme-Linked Immunosorbent Assay (ELISA). Serum cortisol was estimated using ELISA kits manufactured by PUREGENE, Cat. No: PG-0008Ho, Genetix Biotech Asia Pvt. Ltd., New Delhi. Serum Serotonin and epinephrine were estimated by using ELISA kits manufactured by Sincere Biotech Co., Ltd., Beijing, Immunoconcept India Pvt. Ltd., Delhi. The concentration of the hormones in the samples was determined at 450 nm optical density and compared with the standard curve. Serum cortisol, serotonin, and epinephrine concentrations were expressed in ng/mL.

### Antioxidant Profile

The antioxidant markers such as reduced glutathione (GSH), Thiobarbituric Acid Reactive Substances (TBARs), and superoxide dismutase (SOD) were estimated during the study. The estimation of GSH was conducted by the method developed by Beutler et al. (18). The optical density was measured at 4 s 12 nm. The estimation of TBARs and SOD were conducted using the standard methods of analysis (19, 20). Reduced glutathione was expressed in mg/dL, TBARs in moles of MDA/mg of protein, and SOD in U/mg of protein.

### Serum Biochemical Parameters

The analysis of serum levels of total protein (g/dL), albumin (g/dL), globulin (g/dL), glucose (mg/dL), total cholesterol (mg/dL), triglycerides (mg/dL), high density lipoprotein (HDL), cholesterol (mg/dL), low density lipoprotein (LDL), cholesterol (mg/dL), alanine aminotransferase (ALT) (IU/L), aspartate aminotransferase (AST) (IU/L), blood urea nitrogen (BUN) (mg/dL), and creatinine (mg/dL) were performed using commercially manufactured kits.

### Interleukin-6 (IL-6)

Serum IL-6 was measured by ELISA-kit, manufactured by Sincere Biotech Co., Ltd. The equine IL-6 was measured spectrophotometrically at a wavelength of 450 nm, and IL-6 was expressed in pg/ml.

### Statistical Analysis

The data were subjected to statistical analysis by applying two-way ANOVA using the Statistical Package for Social Sciences (SPSS, version-25). For the ANOVA test, the data obtained from the experiment was divided into four groups (one control and three experimental). All hematological data collected on 4 different days were recorded and segregated. Two-way ANOVA was conducted to examine the differences between the means. Differences between means were tested using Duncan's multiple comparison test and the significance level allowed was 0.05. Besides, pair-wise Wilcoxon Signed-Rank *T*-tests were conducted for the day-wise outcome comparison and comparisons of group-specific results.

## Results

**Figure 1:**
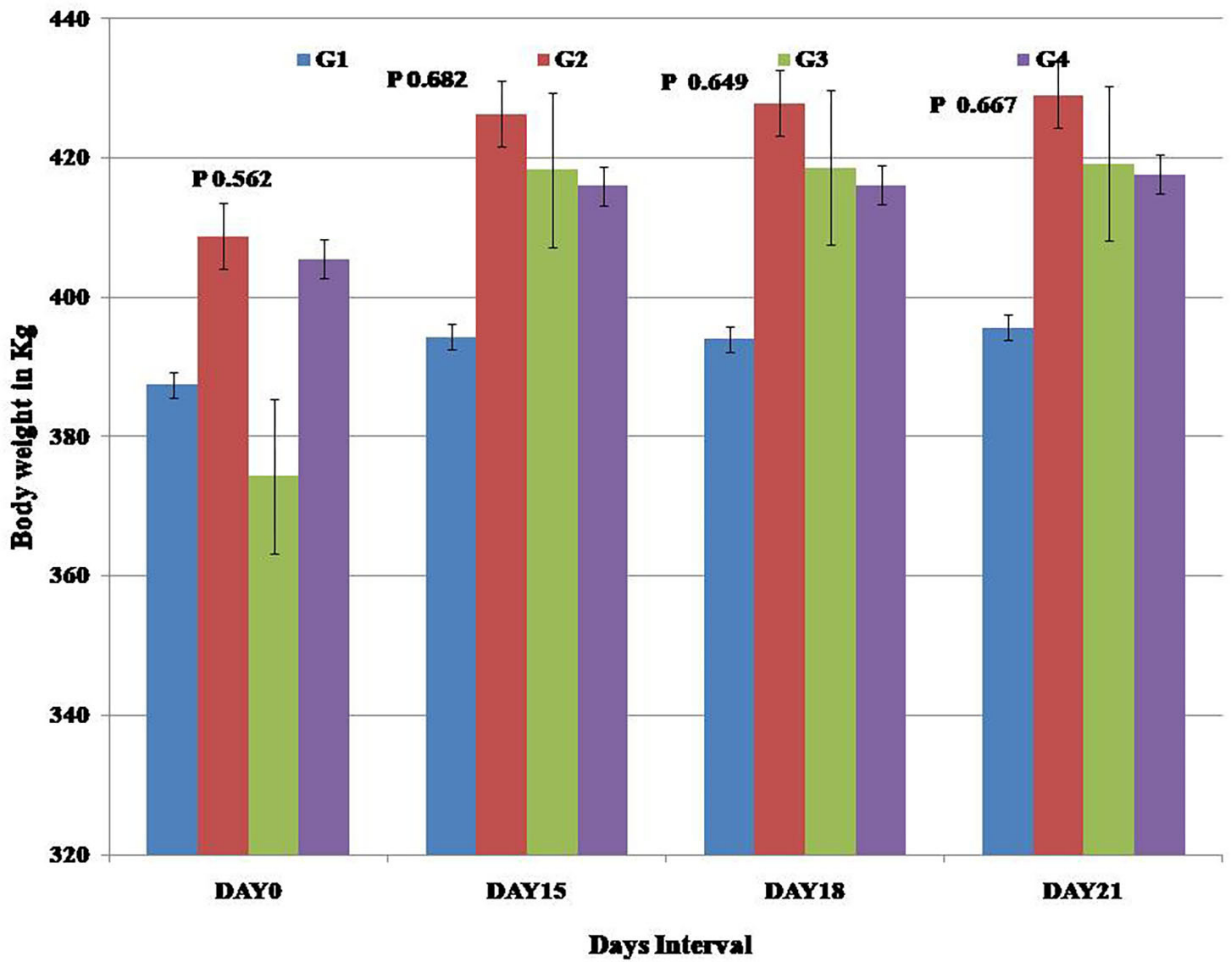
Presentation of body weight (kg) recorded for the horses during the experiment.

All the horses remained healthy throughout the study. There were no deviation from the normal values was reported. At the end of the study, a non-significant increase in the mean body weight of the horses in the treatment groups was observed in comparison to the control group (Figure 1).

### Hematological Parameters

**Figure 2:**
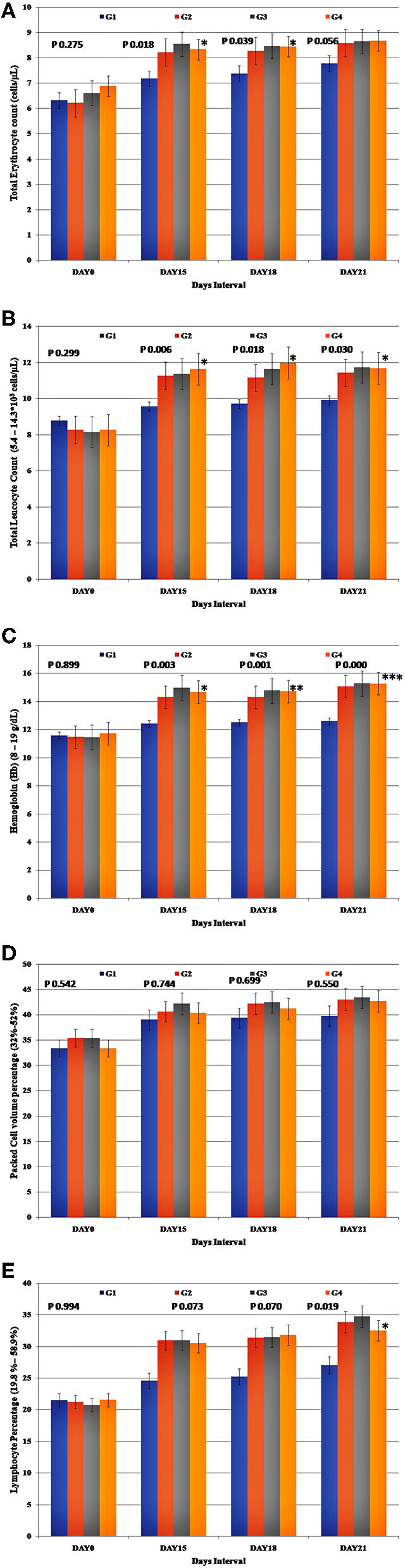
Presentation of the hematological parameters recorded for each study group. **(A)** Group-specific total Erythrocyte count (TEC), **(B)** total Leucocyte count (TLC), **(C)** estimated Hemoglobin (Hb), **(D)** Packed Cell Volume, and **(E)** Lymphocyte percentage.

The mean concentrations (±SE) of hematological markers assessed at Day 0, Day 15, Day 18, and Day 21 in the control and the experimental groups are shown in Figure 2. All the hematological parameters remained within the reference intervals throughout the study in all the experimental groups.

The mean values for the total erythrocyte count (*p* < 0.05), total leukocyte count (*p* < 0.05), hemoglobin (*p* < 0.05), and lymphocyte percentage (*p* < 0.05) were significantly higher in the experimental groups at the end of the study compared to the control group. The increase in the packed cell volume was not significant (*p* < 0.05) in comparison to the control group. There was an increase in the total erythrocyte count, total leucocyte count, hemoglobin, and lymphocyte percentage values in the treatment group 3 (G3) (Figure 2), though not statistically significant, the obtained values were higher than the other treatment groups (G2 & G4).

### Hormonal Profile

**Figure 3:**
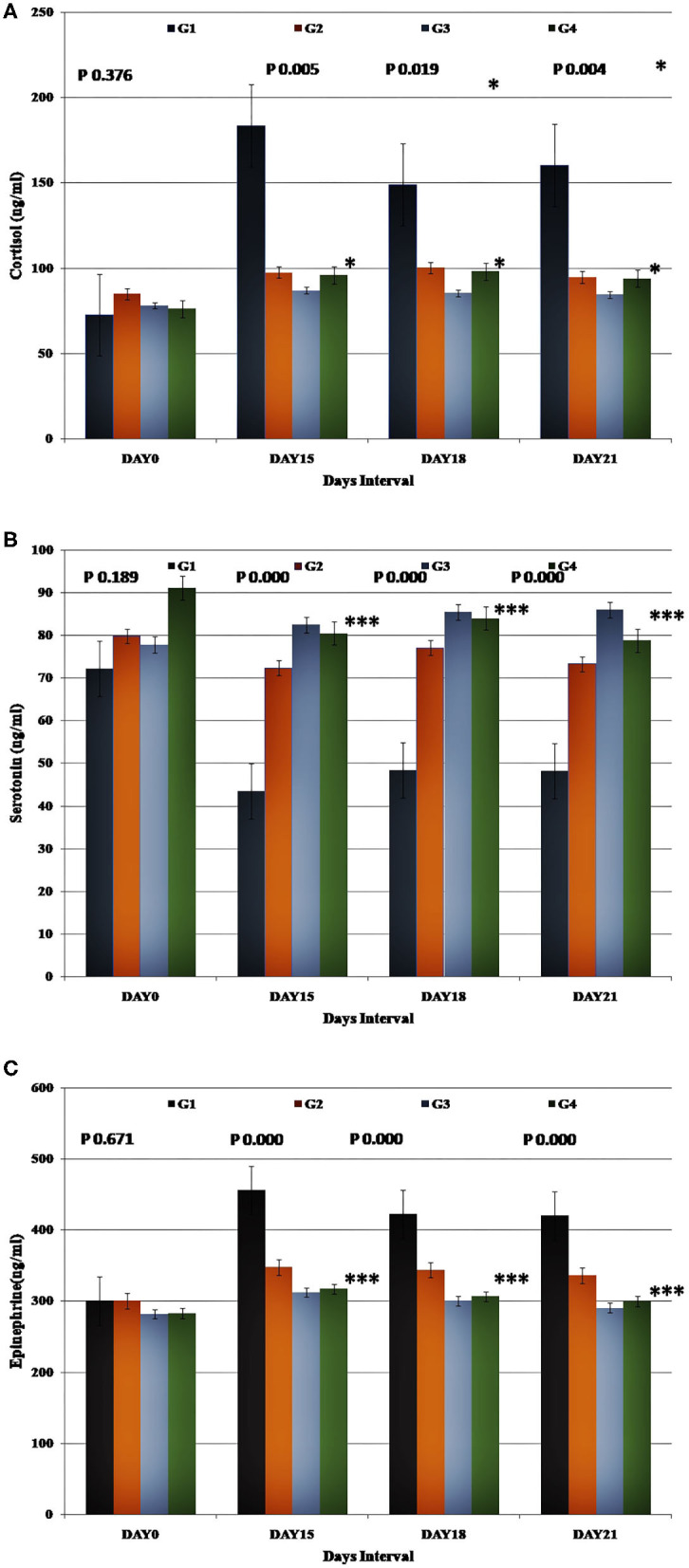
Presentation of the endocrinological parameters recorded for each study group. Group-specific results obtained for **(A)** cortisol, **(B)** serotonin, and **(C)** epinephrine.

Stress hormonal levels in the control and experimental groups were assessed on all the days considered for data recording (Day 0, 15, 18, and 21) and expressed as Mean ± SE (Figure 3). A statistically significant reduction (*p* < 0.05) in the cortisol and epinephrine levels were observed in the experimental groups compared to the control group.

The decrease in the mean values of cortisol (*p* < 0.05) and epinephrine (*p* < 0.05) and the increase in the mean serotonin levels (*p* < 0.05) were significantly higher in the experimental groups (G2, G3, and G4) than the control group.

### Antioxidant Profile Assessment

The mean activity of GSH (mg/dL) and SOD was estimated after the stress induction on the 15th, 18th, and 21st days. The control group displayed significantly (*p* < 0.05) lower values than the baseline. However, in the Ashwagandha treated groups (G2, G3, and G4), the values increased significantly (*p* < 0.05) compared to the control group at the respective time intervals. This signified the effectiveness of the supplement provided to the animals. Besides, the Ashwagandha (WS) treated groups (G2, G3, and G4) displayed significantly better outcomes (*p* < 0.05) compared to their respective baseline records at the post-stress condition. The GSH activity in the experimental group 3(G3) showed better improvement in the outcome on the 21st day. However, the outcome was statistically non-significant compared to the other WS treated groups (G2 and G4).

**Figure 4:**
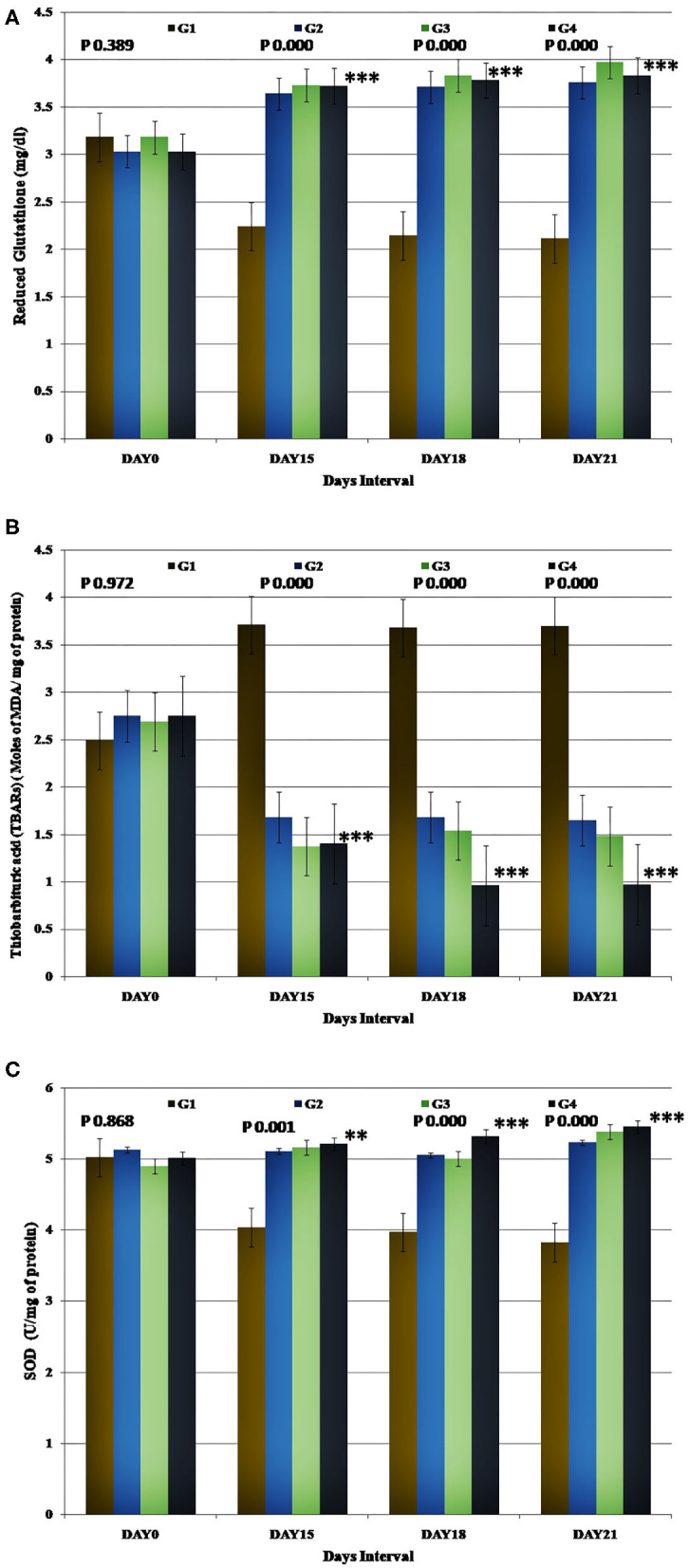
Representation of the antioxidant assessment results for each study group. Group-specific results obtained for **(A)** Reduced Glutathione (GSH), **(B)** Thiobarbituric acid (TBARs), and **(C)** SOD.

The mean concentration of TBARs (moles of MDA/mg of protein) observed during the post-stress on Day 15, 18, and 21 was significantly higher (*p* < 0.05) than the baseline record in the control group. In Ashwagandha treated groups (G2, G3, and G4), a statistically significant (*p* < 0.05) decrease in the TBARs concentration was observed when compared to the control group at the respective time intervals. Relative to the baseline, there was a statistically significant decrease in the concentration of TBARs during the post-stress period in the Ashwagandha treated groups. Though statistically non-significant, the TBARs assessment results in the experimental group 3 (G3) showed a remarkable reduction in comparison to other WS treated groups (G2 and G4) (Figure 4).

### Biochemical Estimations

**Figure 5:**
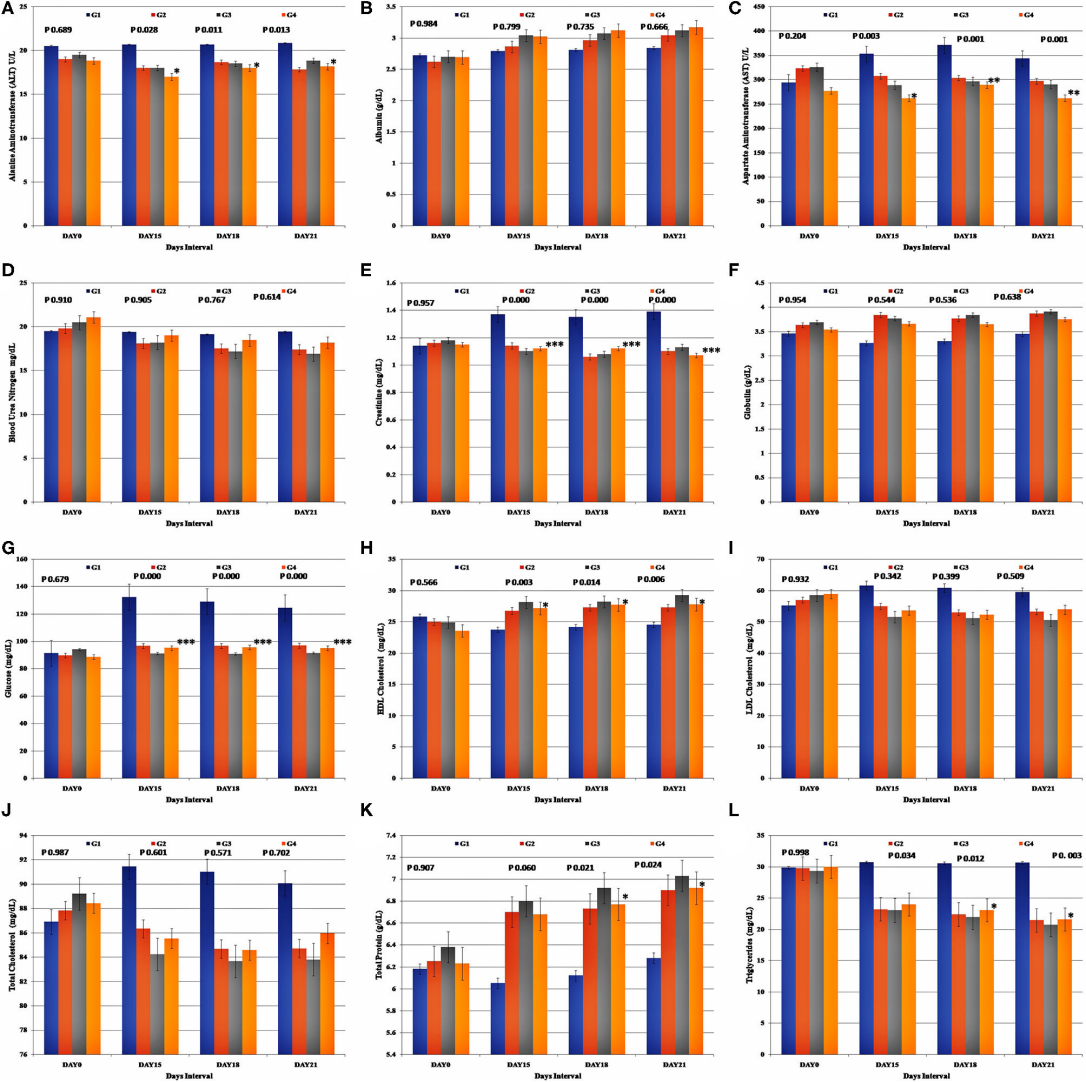
Representation of the biochemical assessment results for each study group. Group-specific results obtained for **(A)** Alanine Aminotransferase (ALT), **(B)** Albumin, **(C)** Aspartate Aminotransferase (AST), **(D)** Blood Urea Nitrogen (BUN), **(E)** Creatinine, **(F)** Globulin, **(G)** Glucose, **(H)** HDL Cholesterol, **(I)** LDL Cholesterol, **(J)** Total Cholesterol, **(K)** Total Protein, and **(L)** Triglycerides.

The biochemical parameters were assessed at the intervals considered (Day 0, 15, 18, and 21) and are expressed by mean (±SE) (Figure 5). All the parameter values recorded, remained within the reference intervals throughout the study in all the study groups.

Relative to the baseline values, the mean values of serum creatinine, glucose, and AST were significantly higher (*p* < 0.05), and the mean values of total cholesterol, LDL cholesterol, HDL cholesterol, triglycerides, total protein, albumin, globulin, ALT, and BUN did not differ significantly relative to the baseline (Day 0) values during the post-stress assessment and at the end of the study in the control group (G1). The increase in the albumin and globulin concentrations and the decrease in the total cholesterol, LDL cholesterol, and BUN in the Ashwagandha treated groups, were not statistically significant when compared to the control group. A significant reduction was observed in the mean values of glucose, ALT (*p* < 0.05), AST (*p* < 0.05), creatinine (*p* < 0.05), and triglycerides (*p* < 0.05), in the treatment groups G2, G3, and G4 than in the control group. Similarly, the mean values of HDL and LDL cholesterol were significantly higher in the Ashwagandha treated groups (G2, G3 and. G4) (*p* < 0.05), whereas the mean concentration of triglycerides was significantly lower in the Ashwagandha treated groups (p < 0.05). On the contrary, assessment of the proteins such as albumin and globulin was not having any significant difference in the experimental group. However, analysis of the lipid content such as total cholesterol, HDL cholesterol, displayed an insignificant decrease in the values for the experimental group. Similar outcomes were noted for the glucose, ALT, AST, creatinine, and BUN estimations for the experimental group. However, the comparative recording of the mean values for triglycerides, HDL cholesterol, glucose, and total protein content showed higher values in the G3 (Group 3) experimental group than the G2 and G4 (Group 2 and Group 4) results.

### Estimation of Immunomodulatory Parameter

#### Interleukin-6 (IL-6)

IL-6 estimation suggested that the mean IL-6 (pg/ml) in the control group during the post-stress assessment on 15th, 18th, and 21st days was comparatively higher and significant (p < 0.05) than baseline. However, in Ashwagandha treated groups (G2, G3, and G4), there were significant (*p* < 0.05) decrease when compared to the control group at respective time intervals. However, the reduction in the IL-6 in the experimental group 3(G3) was comparatively more than other experimental groups (G2 and G4) (Table 1), though the outcome was not statistically significant.

**Table 1:** Assessment outcome of Interleukin-6 (IL-6) for the four horse groups.

**Groups**	**Baseline**		**Post stress**					
	**Day 0**		**Day 15**		**Day 18**		**Day 21**	
	**Mean**	**Standard error**	**Mean**	**Standard error**	**Mean**	**Standard error**	**Mean**	**Standard error**
**Interleukin−6: (pg/ml)**								
**G1**	**133.77**	24.76	**221.11**	19.9	**191.44**	32.21	**224.77**	32.65
**G2**	**144.22**	11.01	**142.72**	13.06	**132.00**	14.73	**135.61**	11.74
**G3**	**142.00**	12.82	**131.94**	7.59	**122.66**	13.29	**129.22**	10.34
**G4**	**152.00**	14.06	142.00^*^	18.68	**137.66**	16.14	146.89^*^	13.94
***p*****-value**	**0.890**		**0.002**		**0.111**		**0.008**	

*The bold format was just to identify the values and it carries no significance*.

Analyses were done to compare all the parameters day-wise following the collection day intervals (Day0, Day15, Day18, and Day21), and group-wise (control group (G1) with all the respective treatment groups (G2, G3, and G4). The obtained differences in the mean value outcomes for each parameter are presented in Supplementary Figures 1–24.

## Discussion

Improvement of animal husbandry and scientific understanding of animal physiology and psychology can yield an effective solution to the problems of the horses. Kathiawari horses were used in the present study, which is a recognized breed from the Saurashtra region of Gujarat state (21). Kathiawari horses have good temperament, strength, pace, and endurance and are genetically close to the Arabian horses. They are characterized by unique inner-curved ear and can survive with nominal rations.

Most drugs used for the physical and mental benefits of the horses have considerable side-effects. For instance, glucocorticoids may persuade suppressed activity of the hypothalamic-pituitary-adrenal axis, and adrenocortical issues (22).

Ashwagandha is used traditionally for humans and animals for health issues and general well-being. “Asvaphalaprakasha” is a documented evidence of Ayurvedic treatment for horses (23). Ashwagandha is having proven anti-inflammatory, anti-cancer, anti-stress, immune-modulatory, antioxidant, adaptogenic, neuroprotective, and beneficial endocrinological activities (24, 25). Ashwagandha roots are having free radical scavenging activity, phenolic compounds, and flavonoids, that are responsible for the antioxidant activity (26). The hepatoprotective property of *Withania somnifera* root powder was proven in rats with significantly lower levels of hepatotoxicity markers including circulatory urea, TBARS, AST, ALT, and ALP (27). No mortality or clinical signs of toxicity in rats were observed even with the maximum recommended dosage of Ashwagandha (28). The anti-stress activity of Ashwagandha and homeostasis management in animals was evaluated in rats through cold swimming stress induction test where better performance was noted (29). Therefore, being an adaptogen and anti-stressor supplement, Ashwagandha could be an ideal supplement choice to manage the physical and mental health conditions of horses.

The present study is the first-ever evaluation conducted in horses to identify and assess the adaptogenic, antioxidant, and immunomodulatory effects of Ashwagandha root extract in varying doses for 21 days. Many important hematological (TEC, TLC, Hb, PCV, Lymphocyte percentage), hormonal (Cortisol, Serotonin, Epinephrine), antioxidant (GSH, TBARs, SOD), biochemical (ALT, Albumin, AST, Blood urea nitrogen, creatinine, globulin, glucose, total cholesterol, HDL, LDL, total proteins, triglycerides), and immunological (IL-6) parameters were assessed. Promising outcomes were obtained.

Separation stress, loud noise, and strenuous exercise can activate the hypothalamic-pituitary-adrenal axis and may induce the secretion of cortisol and epinephrine. The stress response increases the cortisol secretion while diminishing the serotonin levels. Further, an increase in leucocyte count occurs probably due to the enhancement of epinephrine hormone that is responsible for the higher count of lymphocyte migration in the peripheral circulation (30). On the contrary, the decrease in serotonin concentration during stress might be due to excessive secretion of cortisol through the HPA axis which metabolizes serotonin by activation of monoamine oxidase A (MAO-A) (31). Glucose enhancement may occur because of the acute stress stimulated hepatic glucose secretion due to the elevated levels of catecholamines and cortisol. An upsurge in glucose during post-stress might be facilitated by the glucocorticoids, which generally exert catabolic effects in an attempt to utilize the energy resources to counter the imposed stressors through upregulated hepatic gluconeogenesis and plasma glucose levels (32, 33).

Endurance and intensive training can elicit oxidative stress and may alter the antioxidant profile in horses (34). During oxidative stress, there is a decrease in the levels of GSH and an increase in the level of TBARs.

Active ingredients present in Ashwagandha can reduce stress-induced damage, and do not influence the normal body functions much (35). Several studies have demonstrated the beneficial effects such as anti-stress, anxiolytic, and antidepressant effects of Ashwagandha in both humans and animal models (36–38). A study done in geriatric dogs with hepatic dysfunction showed a significant increase in total protein concentration after 14 days of treatment with *Withania somnifera* extract (39). A significant reduction in the pro-inflammatory cytokine IL-6 level in arthritic rats because of WS administration was reported (40). Also, a significant increment in hemoglobin concentration, RBC count, WBC count, platelet count, and body weight was observed in Ashwagandha-treated mice (41). Our observations on the horses are in accordance with these observations.

A similar study has been conducted by Gupta et al. (42) where thyroid hormonal profile and hematological parameters were reported for the Kathiawari horse. The effect of the animal gender was reported for the changes in total erythrocyte counts, MCHC, and MCH in horses. However, the present study reports the impact of the treatment product in the management of stress in comparison to the control animal group. All the animals received the test product was normal and active in behavior during the physical evaluation after each post-stress evaluation session, and also at the end of the study.

The results of the present study indicated a significant reduction in cortisol, epinephrine, glucose, ALT, AST, creatinine, TBARs, triglycerides, and IL-6 whereas considerable increment was observed in the TEC, TLC, Hb, LP, GSH and SOD values. Thus, reduced cortisol and epinephrine levels in the experimental group horses suggest the successful anti-stress effect of Ashwagandha. On the other hand, an increase in the hematological parameters reflects the hematopoietic effect of Ashwagandha in the experimental animals. An upsurge of GSH and SOD values and a decrease in TBARs reflected the better antioxidant level management capability of the herbal supplement.

The decrease in the AST and ALT and creatinine values demonstrated the safety and tolerability of the Ashwagandha root extract by the animals. The reduced level of IL-6 demonstrated the anti-inflammatory impact of Ashwagandha.

## Conclusions

The outcomes of the present study suggested that Ashwagandha root extract has potential hemopoietic, anti-stress, antioxidant, adaptogenic, and immunostimulant properties. The supplement was found safe for the horses. No adverse event was witnessed during the study period for any animal consuming the experimental herbal product. The obtained beneficiary effect observed could be further utilized for the benefit of the horses and could be used to improve or maintain the physical and psychological health of the animal. A large scale study should be conducted in various breeds, different environmental conditions, and considering additional parameters to confirm the present observations.

## Data Availability Statement

The datasets generated for this study are available on request to the corresponding author.

## Ethics Statement

The animal study was reviewed and approved by Institutional Animal Ethics Committee (IAEC) at the College of Veterinary Science, P.V.N.R. Telangana State Veterinary University, Hyderabad (I/2018-38/IAEC/C.V.S., Hyderabad) and Committee for Control and Supervision of Experiments on Animals (CPCSEA), New Delhi, India.

## Author Contributions

GP conducted all the experiments along with BA, ML, and VM. All datasets were inspected and analyzed by BA, ML, and VM. The manuscript was prepared by the authors under the supervision of BK. BK supervised the overall projects. All authors provided their approval for the final version of the article.

## Conflict of Interest

The authors declare that the research was conducted in the absence of any commercial or financial relationships that could be construed as a potential conflict of interest.
