# Supplementary material for: Adaptogenic and Immunomodulatory Activity of Ashwagandha Root Extract: An Experimental Study in an Equine Model
Source: Front Vet Sci. 2020 Sep 29;7:541112. doi: 10.3389/fvets.2020.541112 (PMC7552885; doi:10.3389/fvets.2020.541112)

**Supplementary Materials:**

Pair-wise non-parametric Wilcoxon signed-rank test was conducted for each case with * Vovk-Sellke Maximum p -Ratio: Based on the p -value, the maximum possible odds in favor of H₁ over H₀ equals 1/(-e p log( p )) for p ≤ .37 (Sellke, Bayarri, & Berger, 2001). Hodges-Lehmann Estimate was used with 95% confidential intervals (CI). A 95% CI for Rank-Biserial Correlation was also estimated.

**Supplementary Figure 1:** Comparative pair-wise T-test (Wilcoxon signed-rank) outcomes for **body weight** measurements taken in different intervals. (A) Representation of comparative values for Day0, Day15, Day18, and Day21. (B) Comparative pair-wise T-test (Wilcoxon signed-rank) outcomes for **body weight** measurements taken for G1 (control), G2, G3, and G4 groups.


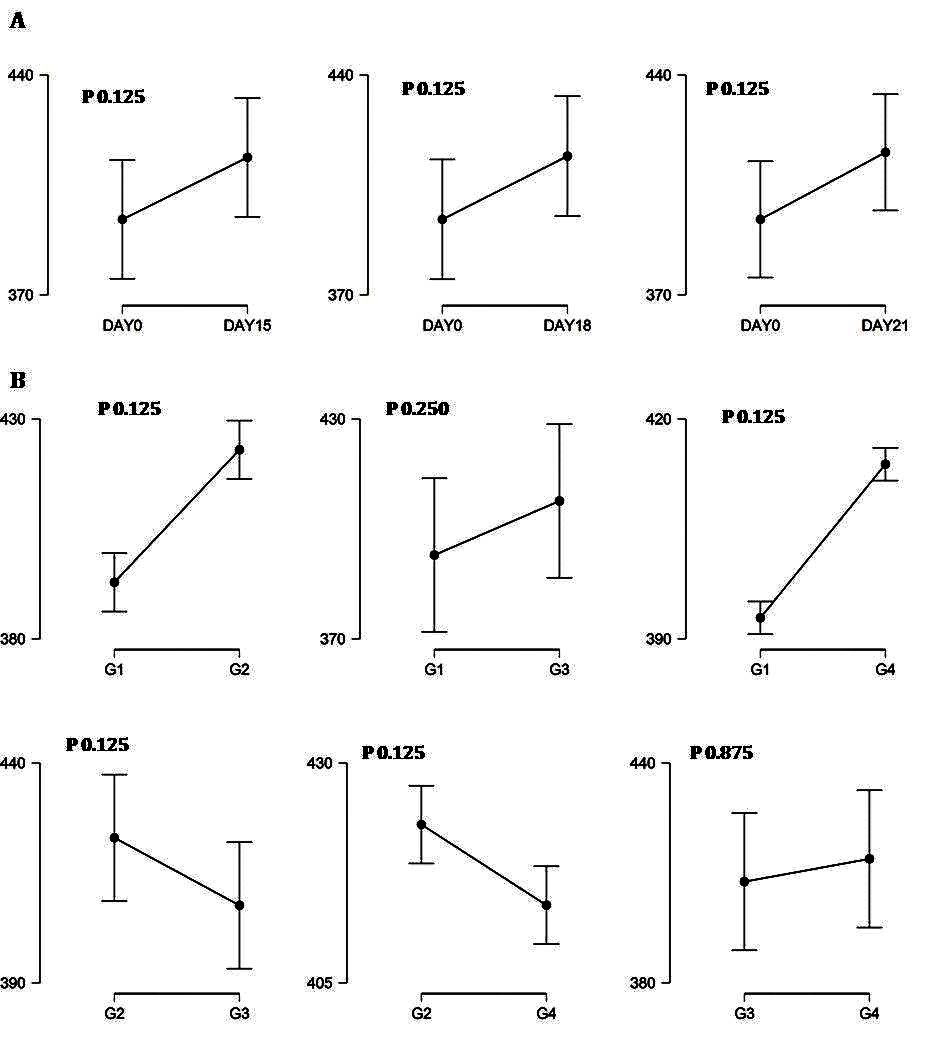


**Supplementary Figure 2:** Comparative pair-wise T-test (Wilcoxon signed-rank) outcomes for **total erythrocyte count** estimations in different intervals. (A) Representation of comparative values for Day0, Day15, Day18, and Day21. (B) Comparative pair-wise T-test (Wilcoxon signed-rank) outcomes for **total erythrocyte count** estimations done for G1 (control), G2, G3, and G4 groups.


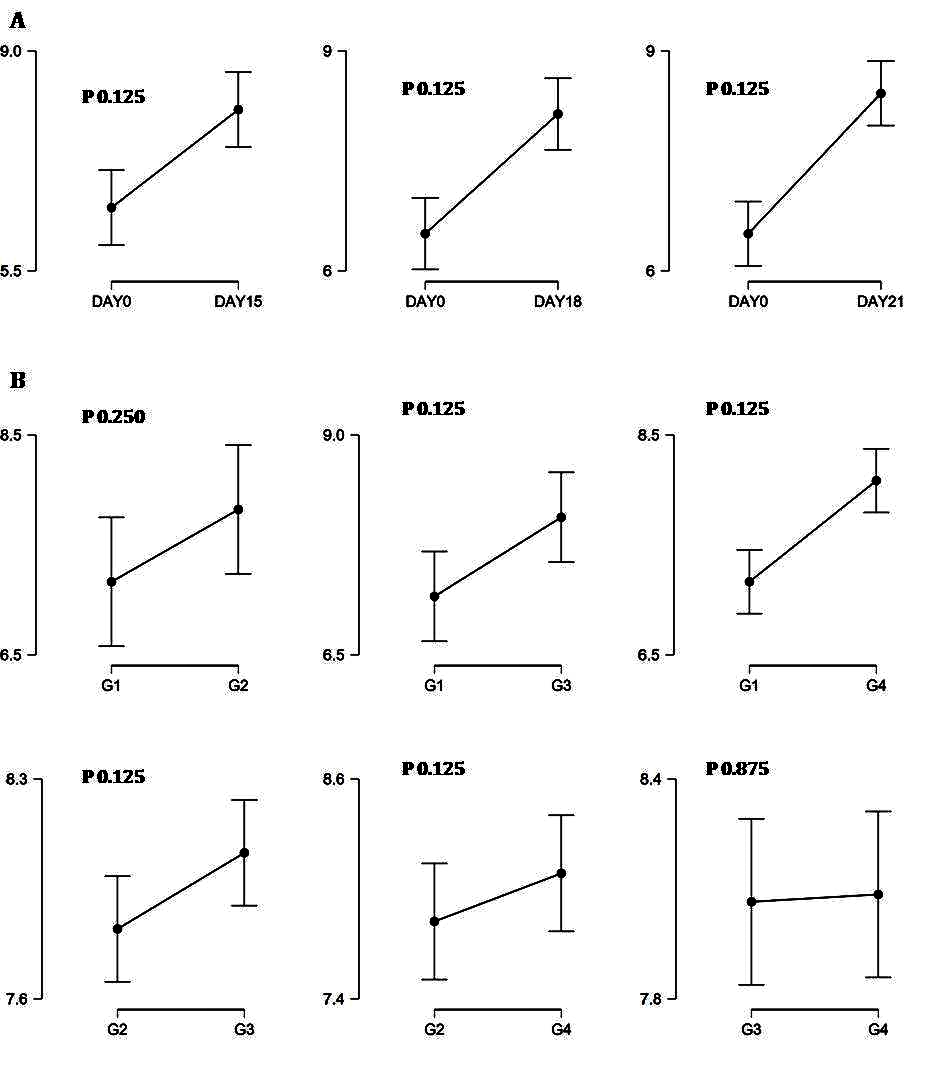


**Supplementary Figure 3:** Comparative pair-wise T-test (Wilcoxon signed-rank) outcomes for t**otal Leucocyte count** estimations in different intervals. (A) Representation of comparative values for Day0, Day15, Day18, and Day21. (B) Comparative pair-wise T-test (Wilcoxon signed-rank) outcomes for **total Leucocyte count** estimations done for G1 (control), G2, G3, and G4 groups.


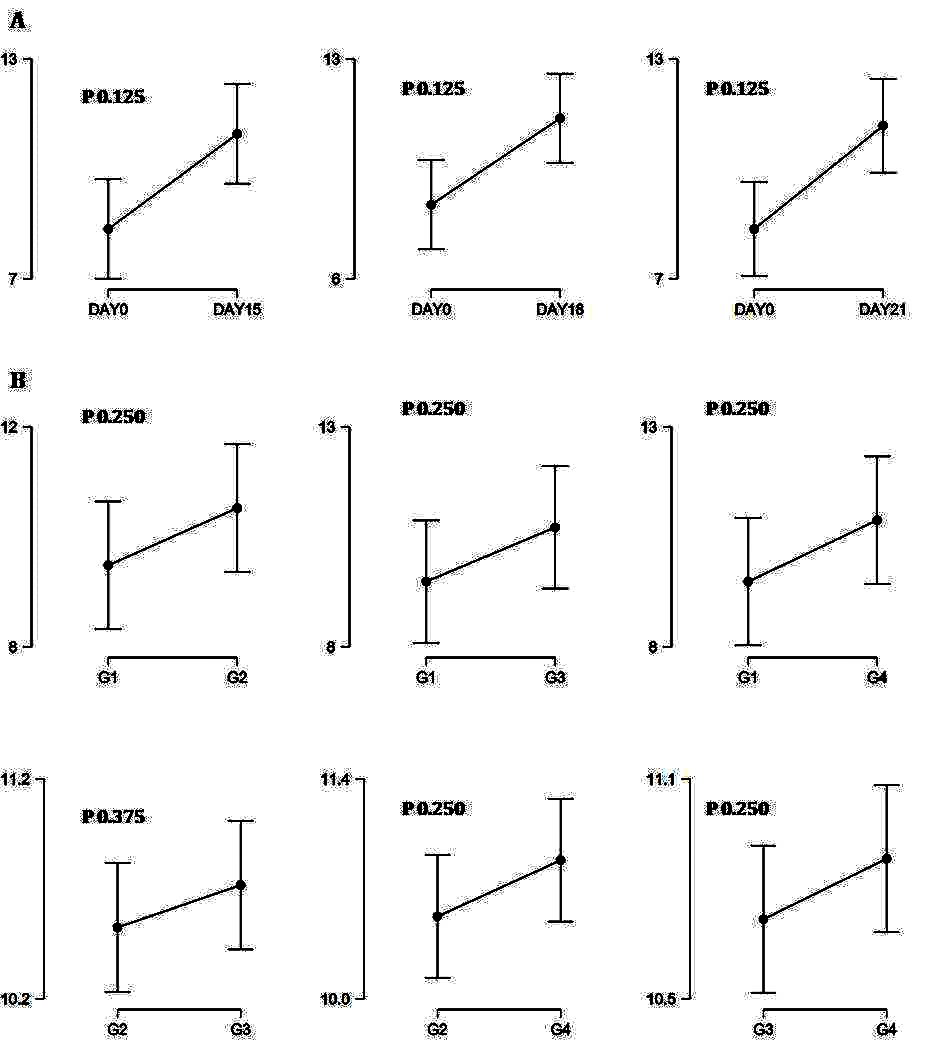


**Supplementary Figure 4:** Comparative pair-wise T-test (Wilcoxon signed-rank) outcomes for **hemoglobin** estimations in different intervals. (A) Representation of comparative values for Day0, Day15, Day18, and Day21. (B) Comparative pair-wise T-test (Wilcoxon signed-rank) outcomes for **hemoglobin** estimations done for G1 (control), G2, G3, and G4 groups.


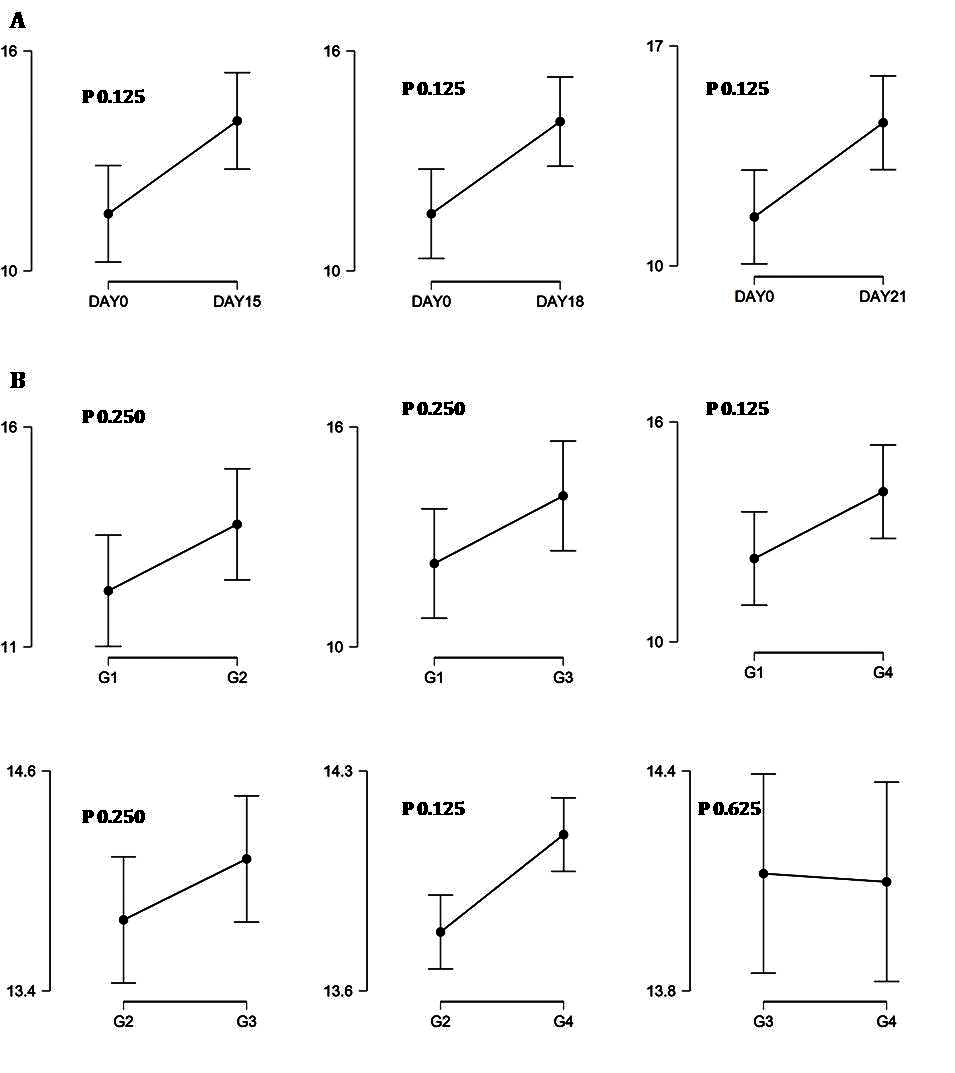


**Supplementary Figure 5:** Comparative pair-wise T-test (Wilcoxon signed-rank) outcomes for **packed cell volume** estimations in different intervals. (A) Representation of comparative values for Day0, Day15, Day18, and Day21. (B) Comparative pair-wise T-test (Wilcoxon signed-rank) outcomes for **packed cell volume** estimations done for G1 (control), G2, G3, and G4 groups.

**
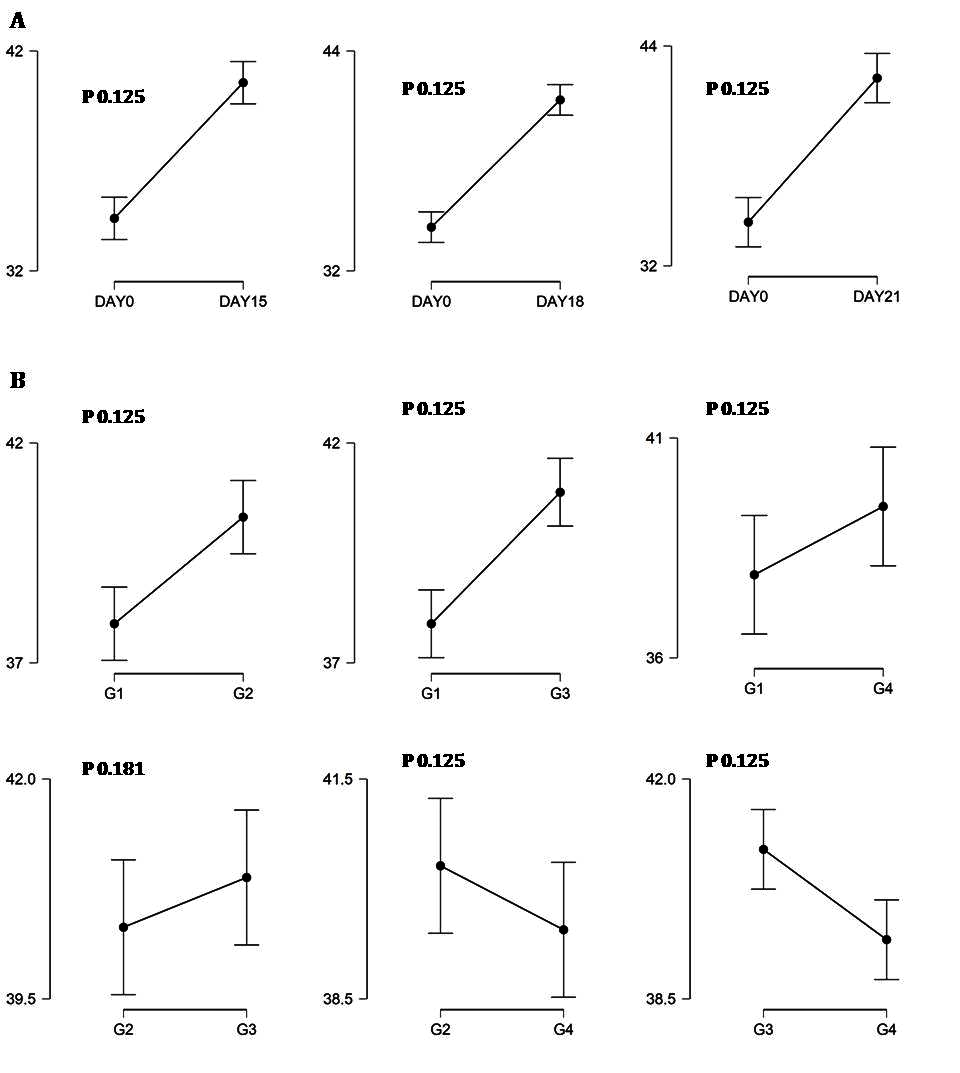
**

**Supplementary Figure 6:** Comparative pair-wise T-test (Wilcoxon signed-rank) outcomes for **Lymphocyte percentage** observed in different intervals. (A) Representation of comparative values for Day0, Day15, Day18, and Day21. (B) Comparative pair-wise T-test (Wilcoxon signed-rank) outcomes for **Lymphocyte percentage** estimations done for G1 (control), G2, G3, and G4 groups.

**
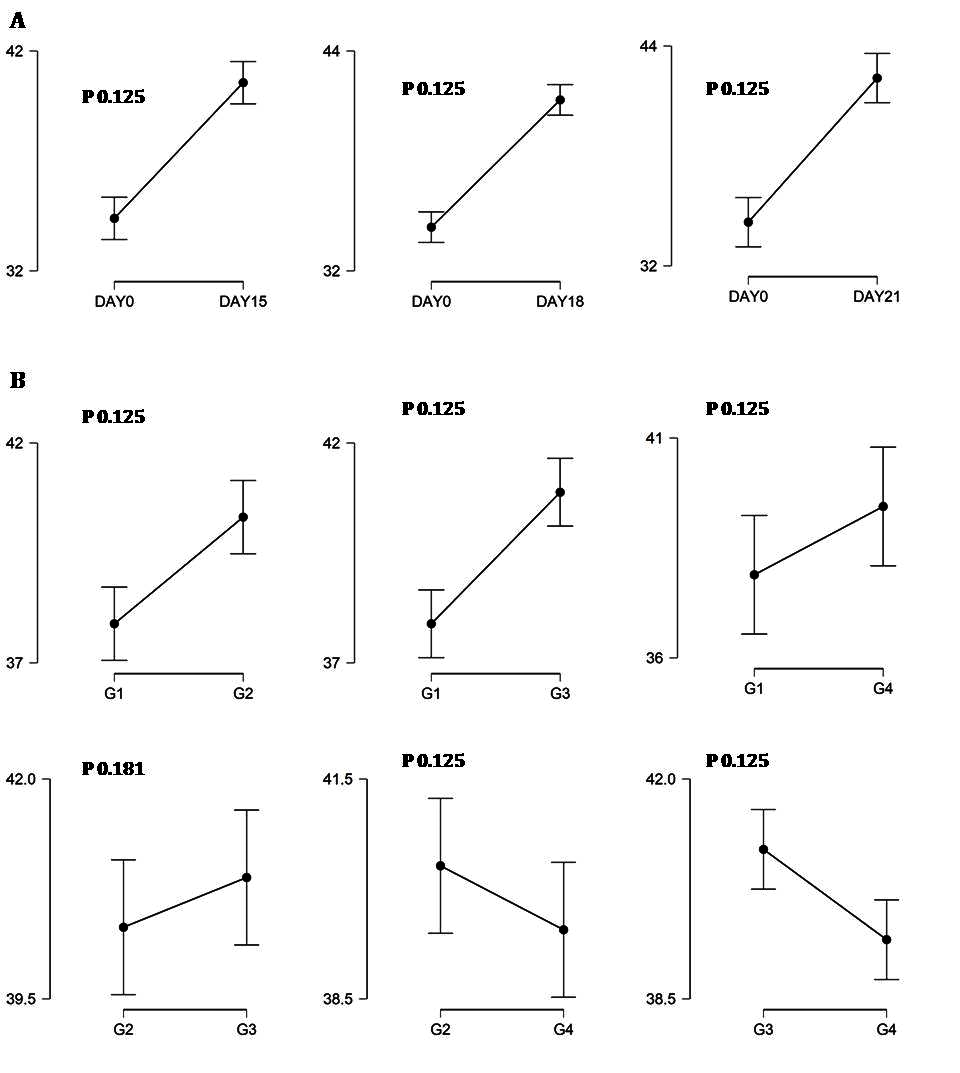
**

**Supplementary Figure 7:** Comparative pair-wise T-test (Wilcoxon signed-rank) outcomes for **Cortisol** estimations in different intervals. (A) Representation of comparative values for Day0, Day15, Day18, and Day21. (B) Comparative pair-wise T-test (Wilcoxon signed-rank) outcomes for **Cortisol** estimations done for G1 (control), G2, G3, and G4 groups.


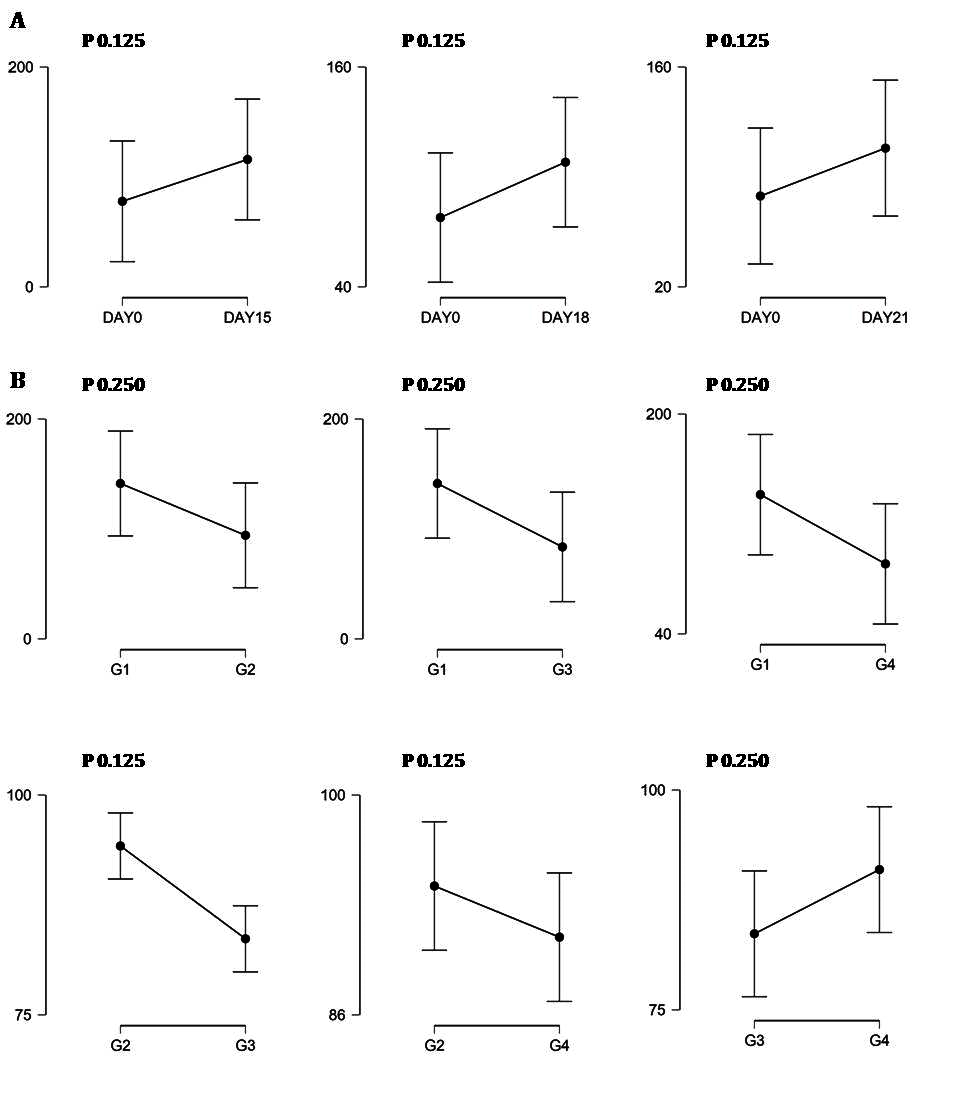


**Supplementary Figure 8:** Comparative pair-wise T-test (Wilcoxon signed-rank) outcomes for **Serotonin** estimations in different intervals. (A) Representation of comparative values for Day0, Day15, Day18, and Day21. (B) Comparative pair-wise T-test (Wilcoxon signed-rank) outcomes for **Serotonin** estimations done for G1 (control), G2, G3, and G4 groups.


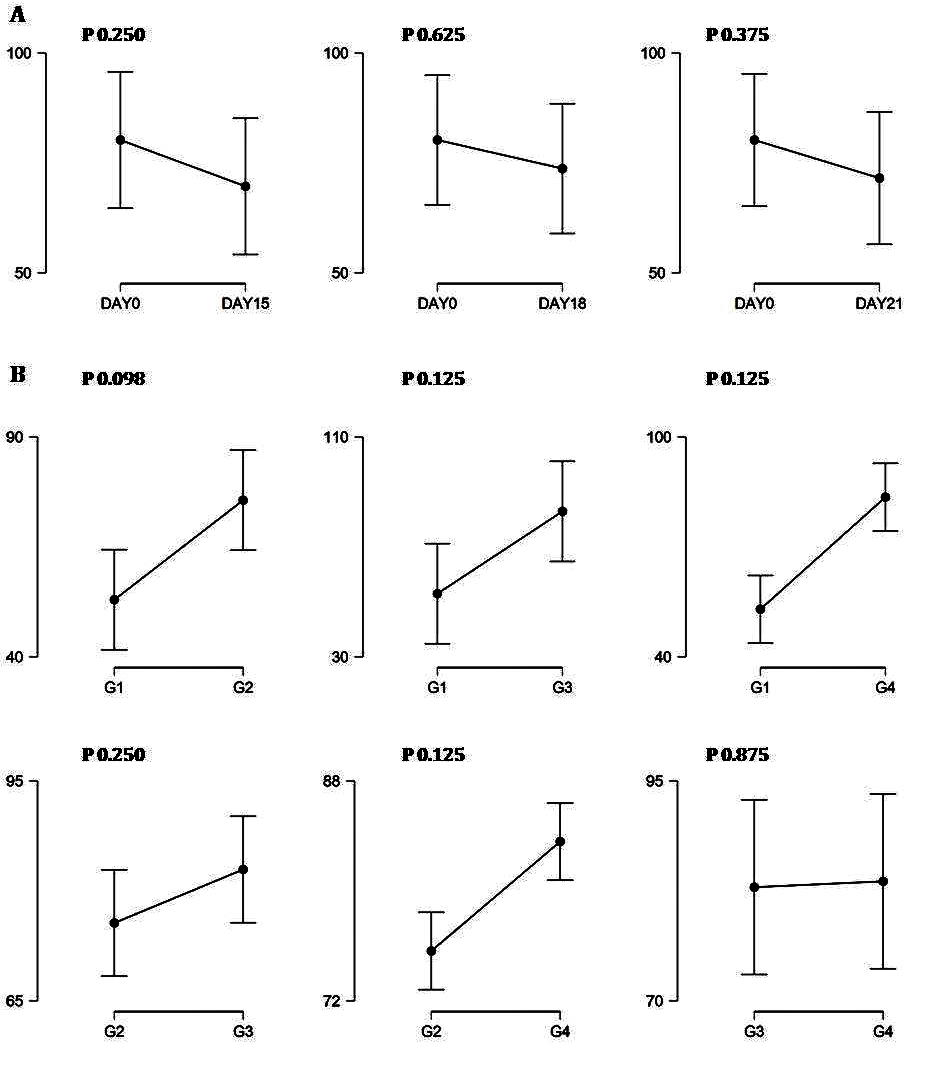


**Supplementary Figure 9:** Comparative pair-wise T-test (Wilcoxon signed-rank) outcomes for **Epinephrine** estimations in different intervals. (A) Representation of comparative values for Day0, Day15, Day18, and Day21. (B) Comparative pair-wise T-test (Wilcoxon signed-rank) outcomes for **Epinephrine** estimations done for G1 (control), G2, G3, and G4 groups.

**
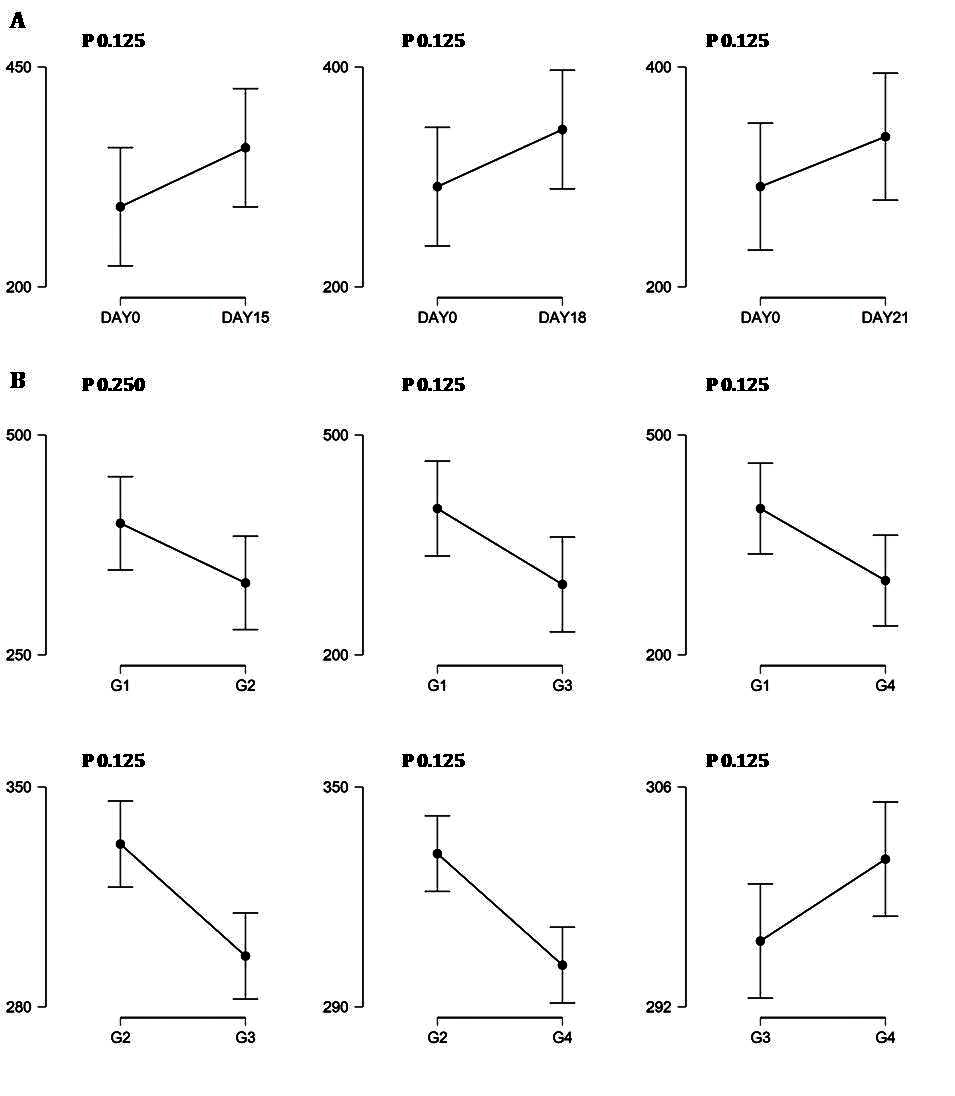
**

**Supplementary Figure 9:** Comparative pair-wise T-test (Wilcoxon signed-rank) outcomes for **reduced Glutathione (GSH)** estimations in different intervals. (A) Representation of comparative values for Day0, Day15, Day18, and Day21. (B) Comparative pair-wise T-test (Wilcoxon signed-rank) outcomes for **reduced Glutathione (GSH)** estimations done for G1 (control), G2, G3, and G4 groups.


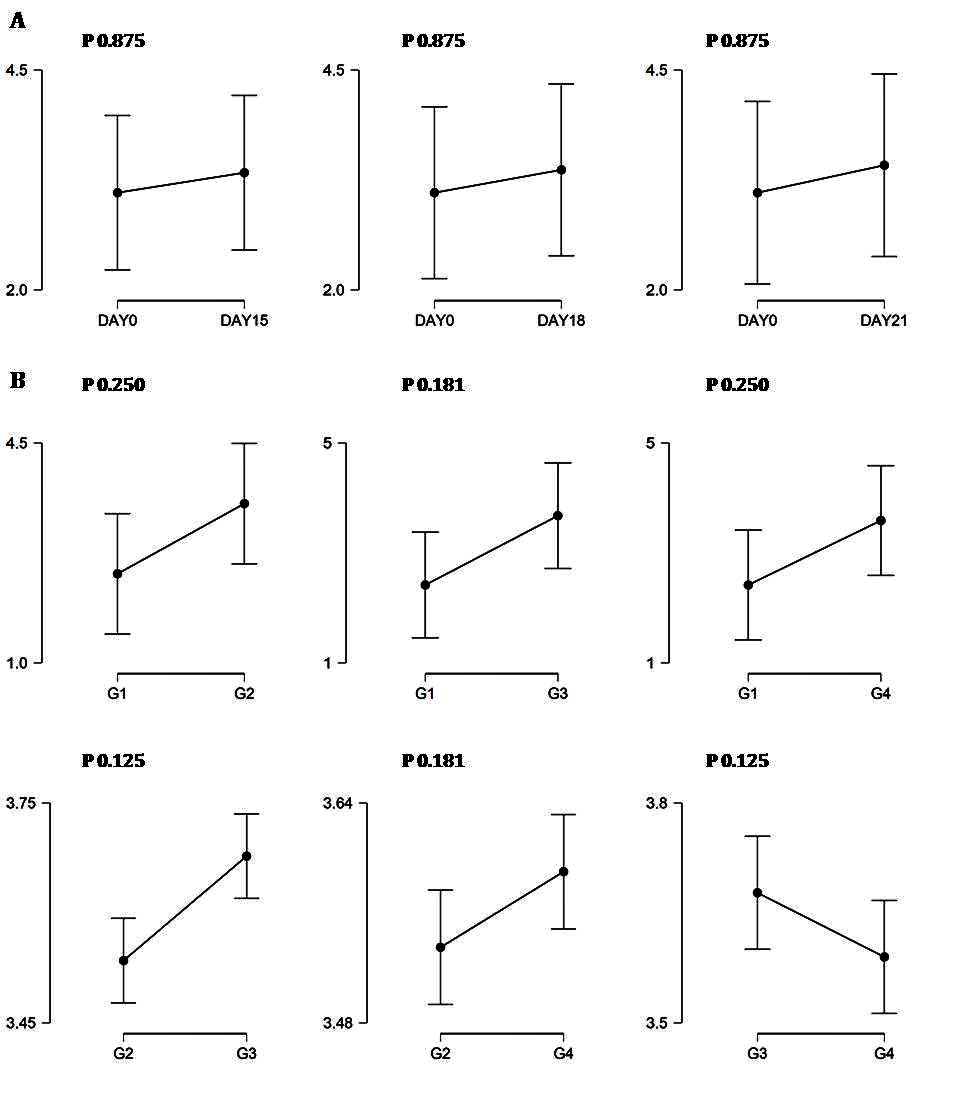


**Supplementary Figure 10:** Comparative pair-wise T-test (Wilcoxon signed-rank) outcomes for **Thiobarbituric acid (TBARs)** estimations in different intervals. (A) Representation of comparative values for Day0, Day15, Day18, and Day21. (B) Comparative pair-wise T-test (Wilcoxon signed-rank) outcomes for **Thiobarbituric acid (TBARs)** estimations done for G1 (control), G2, G3, and G4 groups.


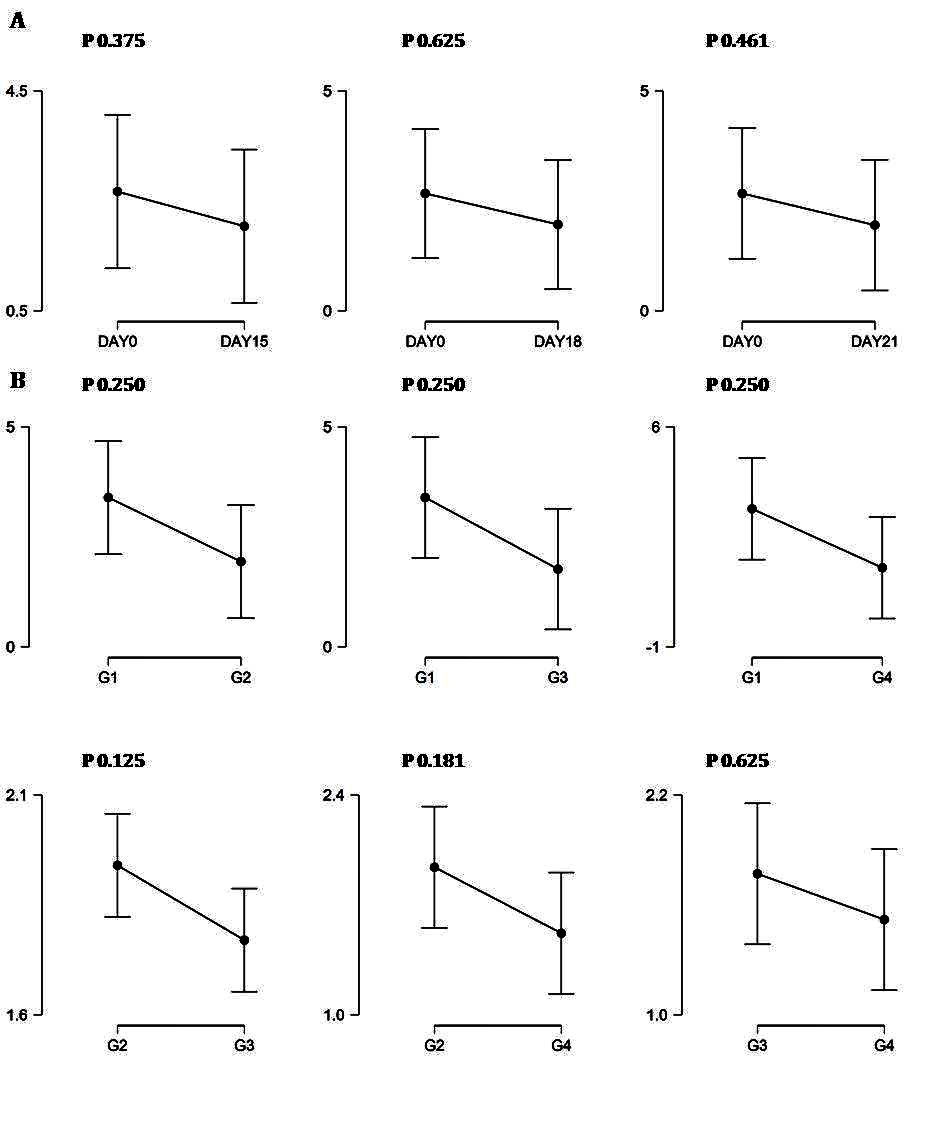


**Supplementary Figure 11:** Comparative pair-wise T-test (Wilcoxon signed-rank) outcomes for **SOD** estimations in different intervals. (A) Representation of comparative values for Day0, Day15, Day18, and Day21. (B) Comparative pair-wise T-test (Wilcoxon signed-rank) outcomes for **SOD** estimations done for G1 (control), G2, G3, and G4 groups.

**
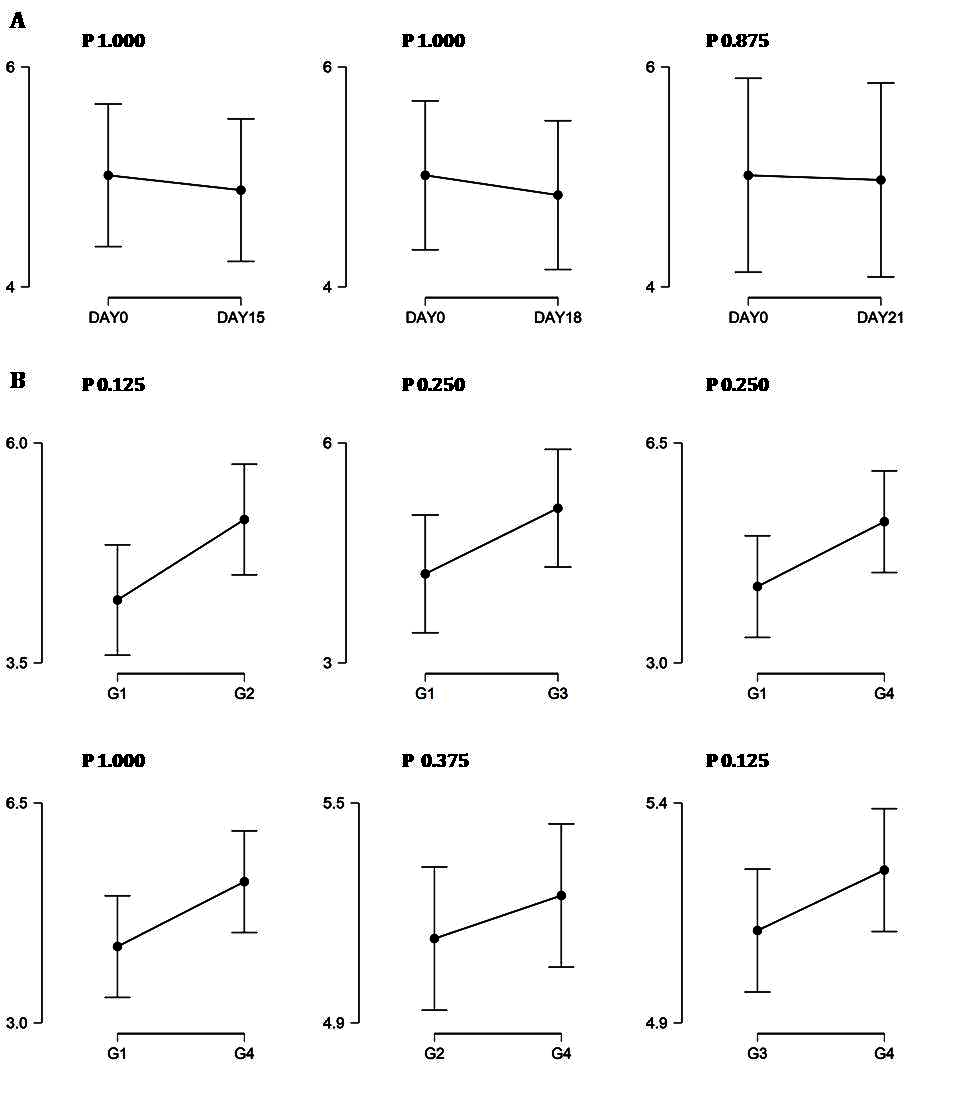
**

**Supplementary Figure 12:** Comparative pair-wise T-test (Wilcoxon signed-rank) outcomes for **Alanine Aminotransferase (ALT)** estimations in different intervals. (A) Representation of comparative values for Day0, Day15, Day18, and Day21. (B) Comparative pair-wise T-test (Wilcoxon signed-rank) outcomes for **Alanine Aminotransferase (ALT)** estimations done for G1 (control), G2, G3, and G4 groups.


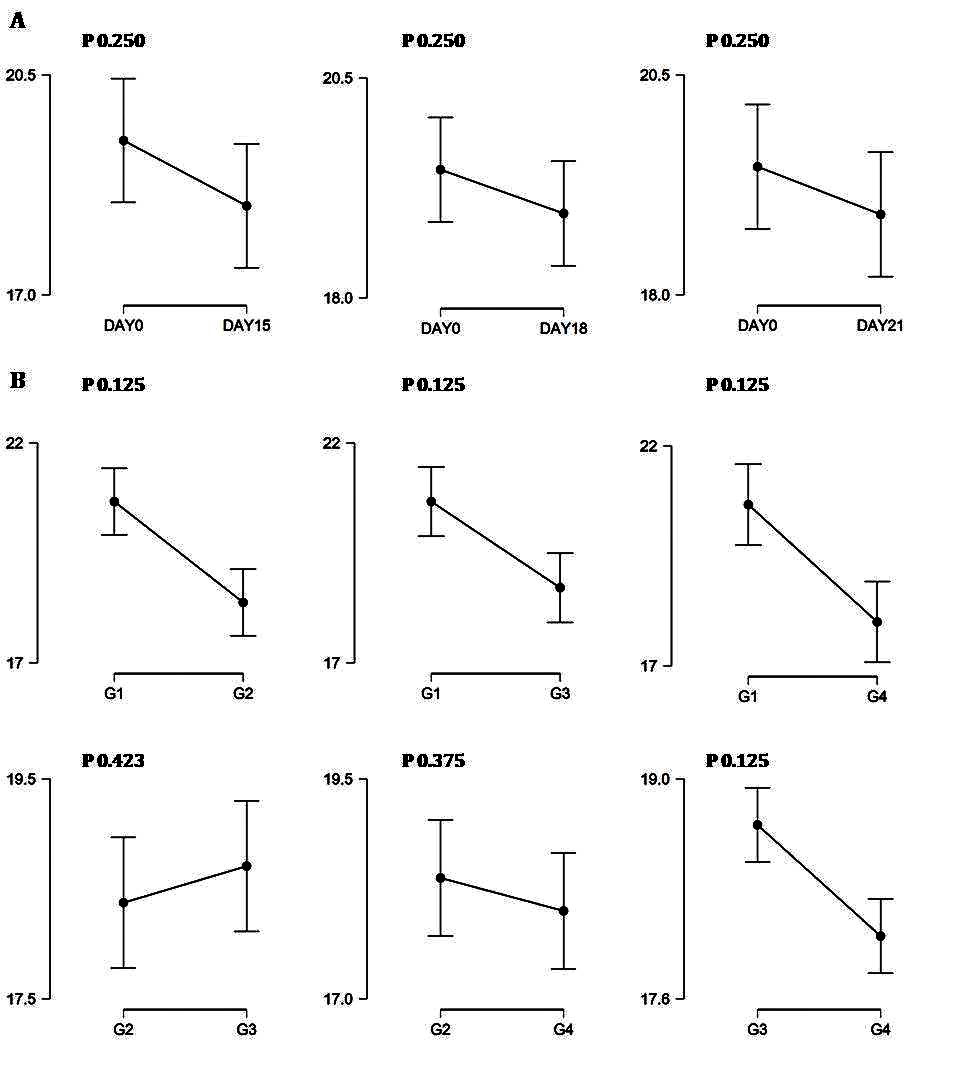


**Supplementary Figure 13:** Comparative pair-wise T-test (Wilcoxon signed-rank) outcomes for **Albumin** estimations in different intervals. (A) Representation of comparative values for Day0, Day15, Day18, and Day21. (B) Comparative pair-wise T-test (Wilcoxon signed-rank) outcomes for **Albumin** estimations done for G1 (control), G2, G3, and G4 groups.

**
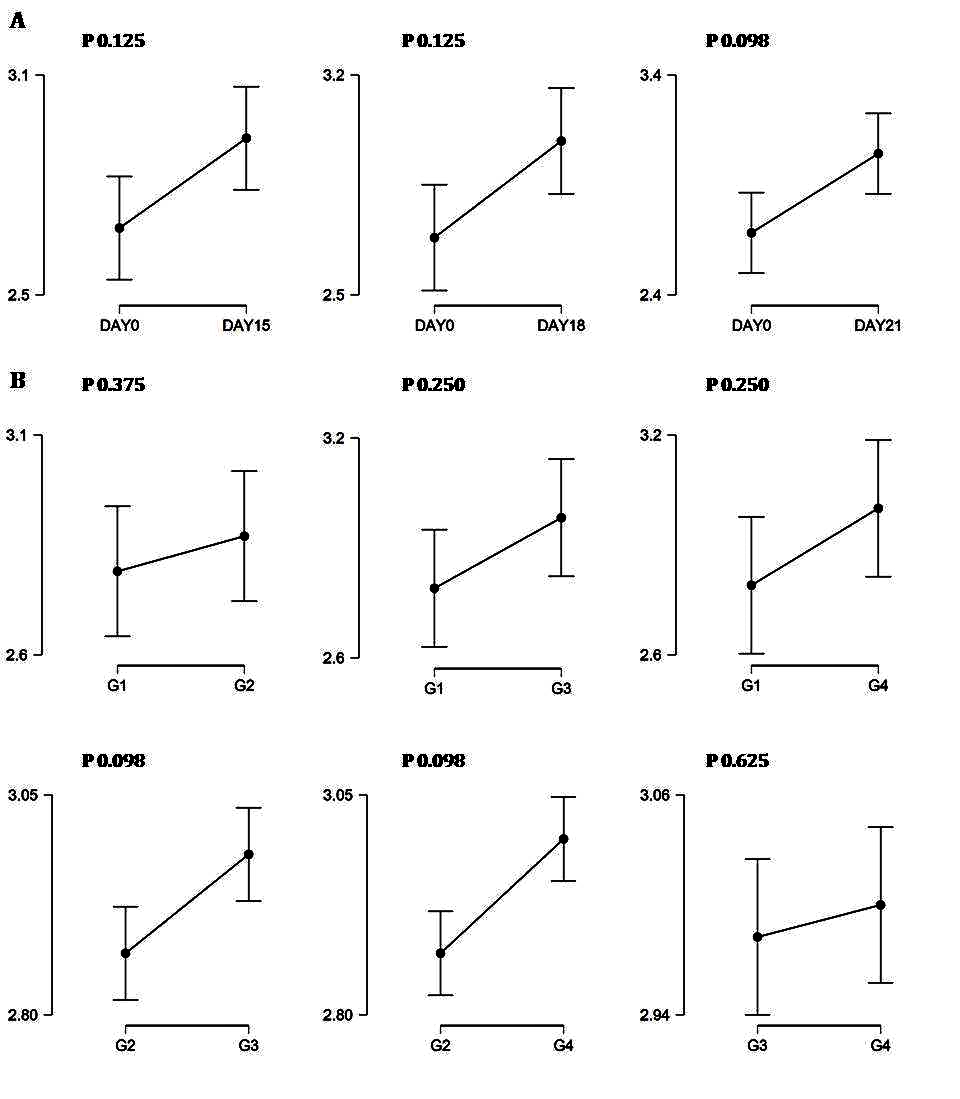
**

**Supplementary Figure 14:** Comparative pair-wise T-test (Wilcoxon signed-rank) outcomes for **Aspartate Aminotransferase (AST)** estimations in different intervals. (A) Representation of comparative values for Day0, Day15, Day18, and Day21. (B) Comparative pair-wise T-test (Wilcoxon signed-rank) outcomes for **Aspartate Aminotransferase (AST)** estimations done for G1 (control), G2, G3, and G4 groups.


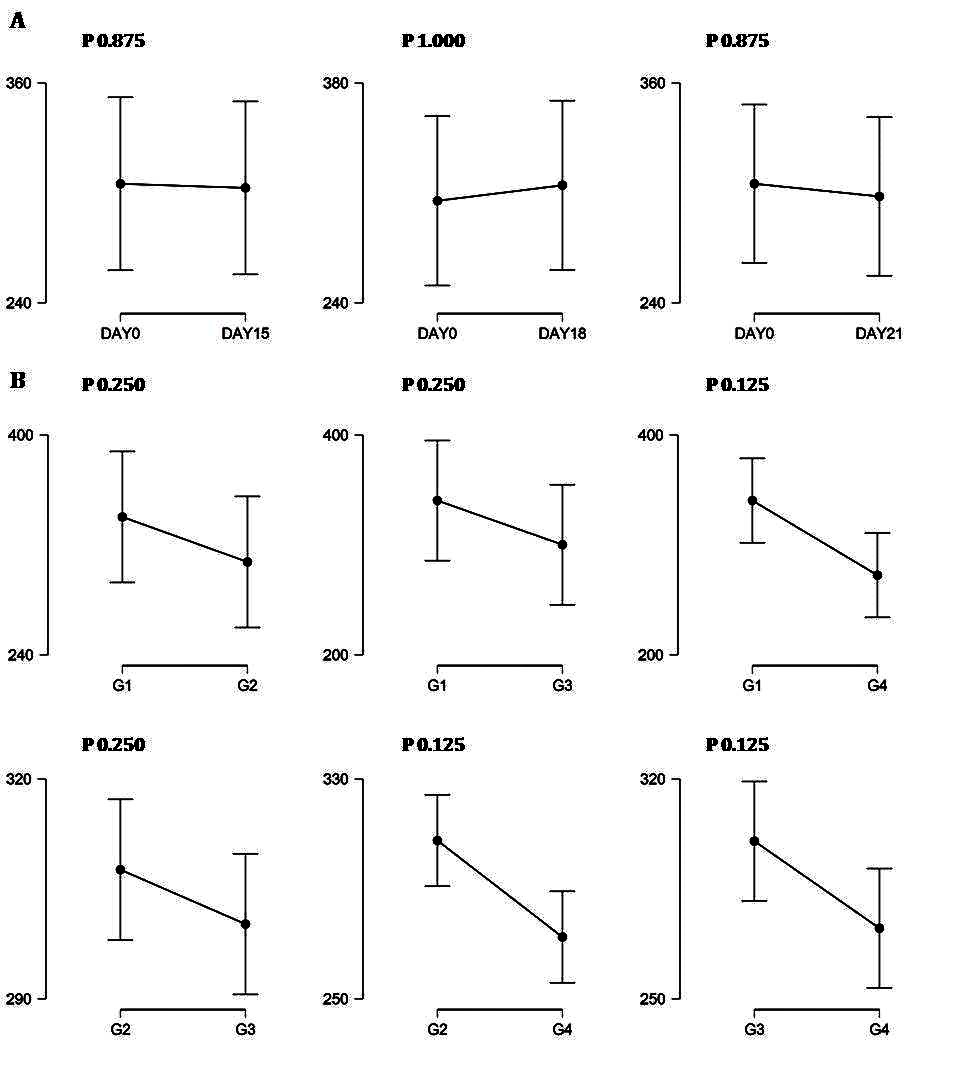


**Supplementary Figure 15:** Comparative pair-wise T-test (Wilcoxon signed-rank) outcomes for **Blood Urea Nitrogen (BUN)** estimations in different intervals. (A) Representation of comparative values for Day0, Day15, Day18, and Day21. (B) Comparative pair-wise T-test (Wilcoxon signed-rank) outcomes for **Blood Urea Nitrogen (BUN)** estimations done for G1 (control), G2, G3, and G4 groups.


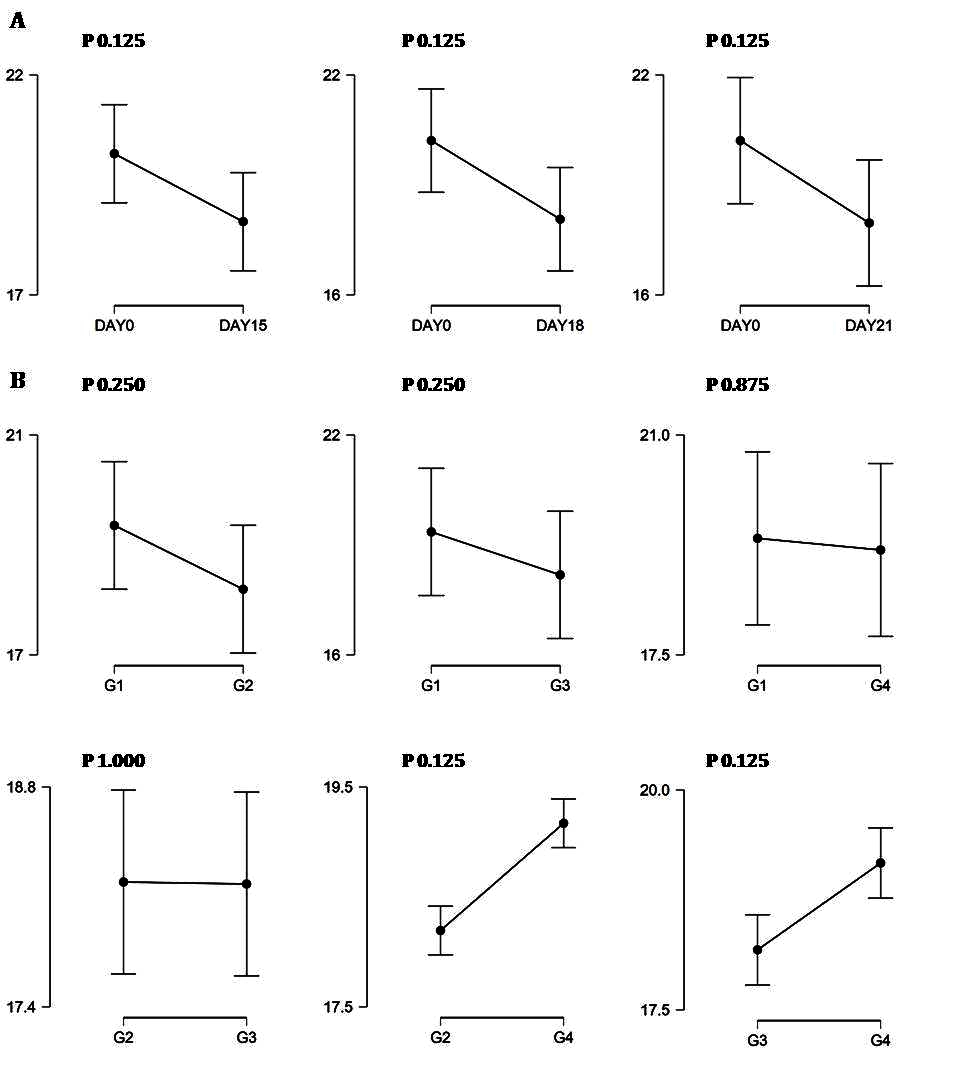


**Supplementary Figure 16:** Comparative pair-wise T-test (Wilcoxon signed-rank) outcomes for **Creatinine** estimations in different intervals. (A) Representation of comparative values for Day0, Day15, Day18, and Day21. (B) Comparative pair-wise T-test (Wilcoxon signed-rank) outcomes for **Creatinine** estimations done for G1 (control), G2, G3, and G4 groups.

**
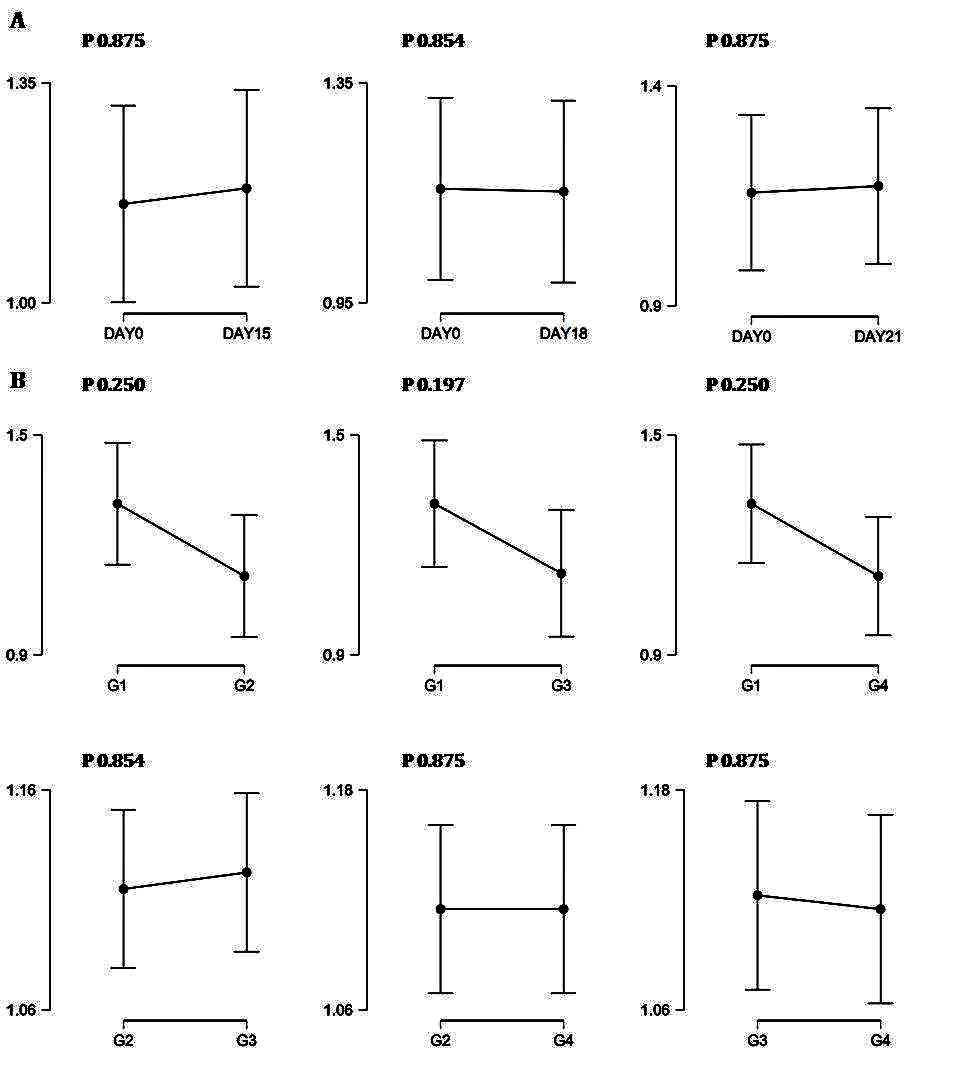
**

**Supplementary Figure 17:** Comparative pair-wise T-test (Wilcoxon signed-rank) outcomes for **Globulin** estimations in different intervals. (A) Representation of comparative values for Day0, Day15, Day18, and Day21. (B) Comparative pair-wise T-test (Wilcoxon signed-rank) outcomes for **Globulin** estimations done for G1 (control), G2, G3, and G4 groups.

**
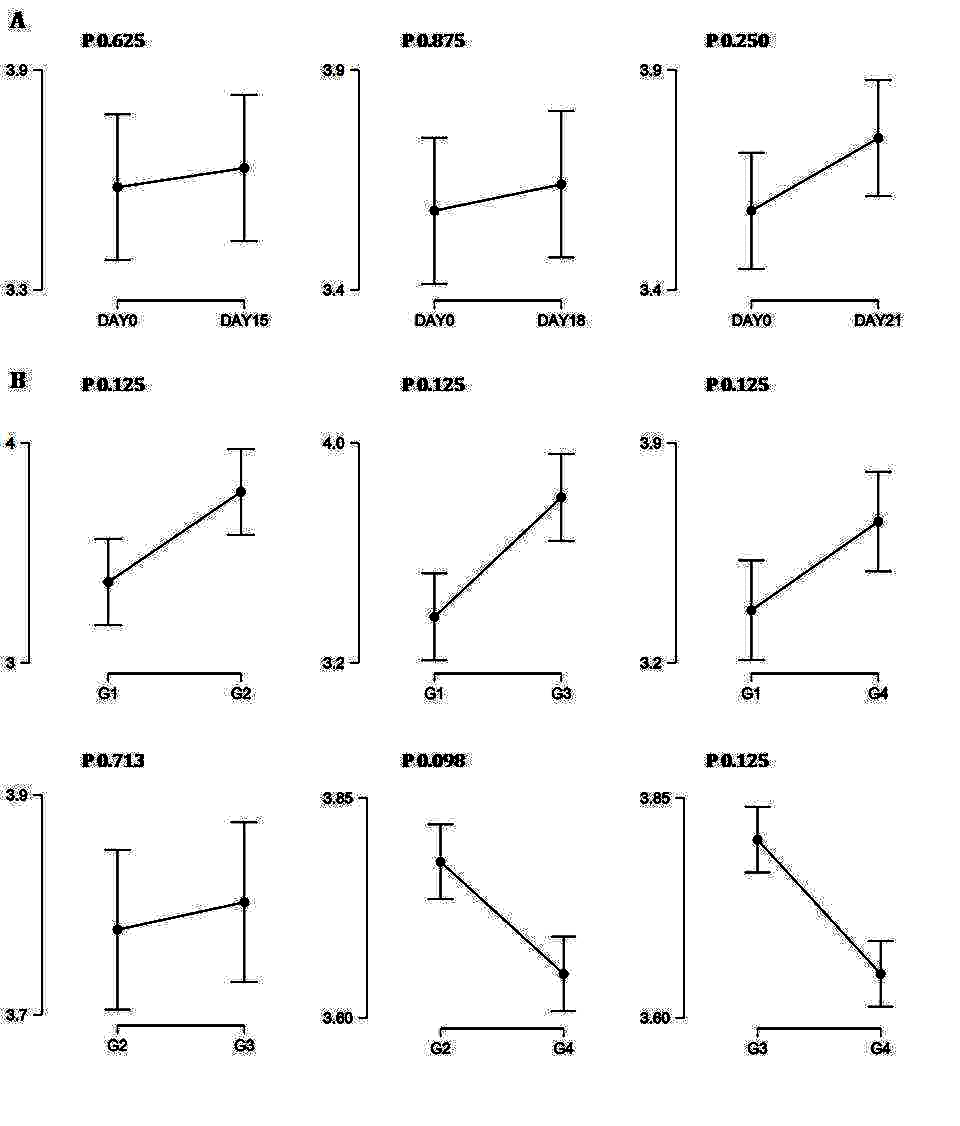
**

**Supplementary Figure 18:** Comparative pair-wise T-test (Wilcoxon signed-rank) outcomes for **Glucose** estimations in different intervals. (A) Representation of comparative values for Day0, Day15, Day18, and Day21. (B) Comparative pair-wise T-test (Wilcoxon signed-rank) outcomes for **Glucose** estimations done for G1 (control), G2, G3, and G4 groups.


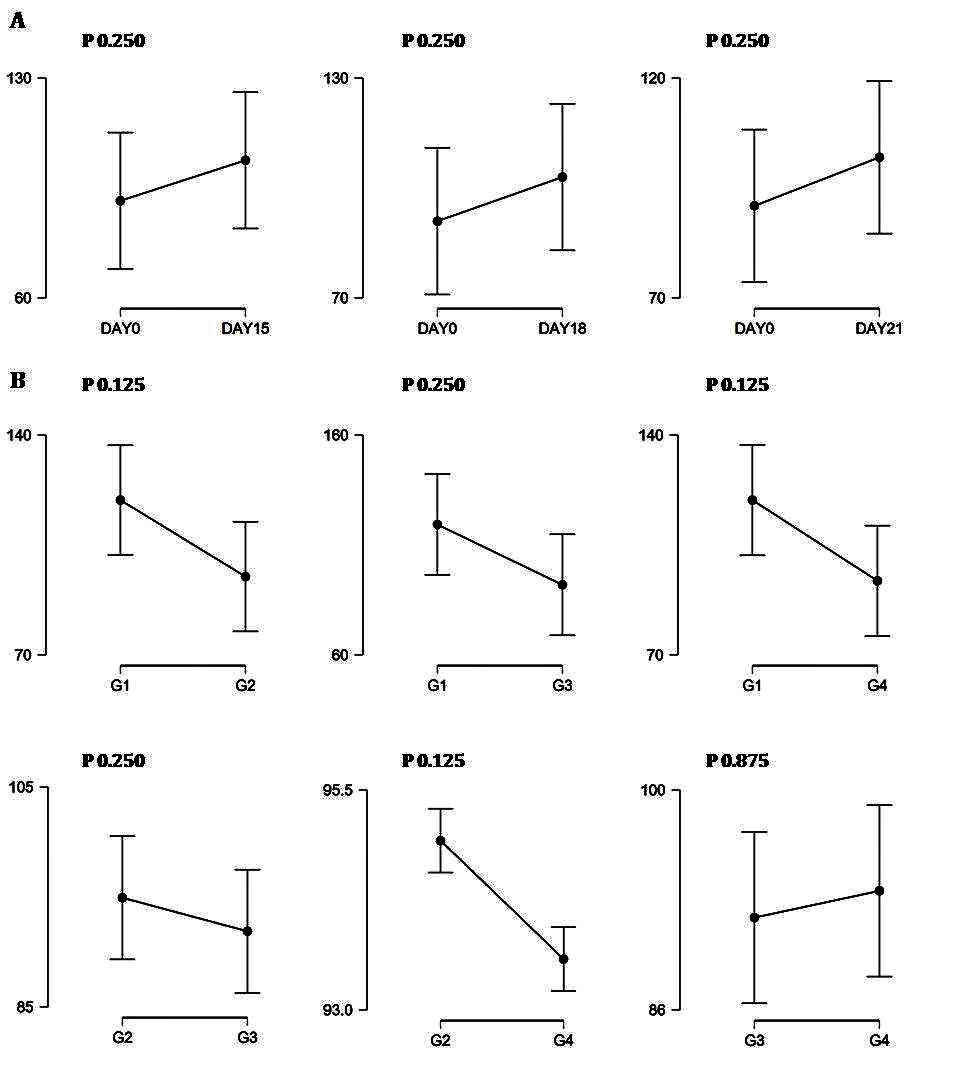


**Supplementary Figure 19:** Comparative pair-wise T-test (Wilcoxon signed-rank) outcomes for **HDL** estimations in different intervals. (A) Representation of comparative values for Day0, Day15, Day18, and Day21. (B) Comparative pair-wise T-test (Wilcoxon signed-rank) outcomes for **HDL** estimations done for G1 (control), G2, G3, and G4 groups.


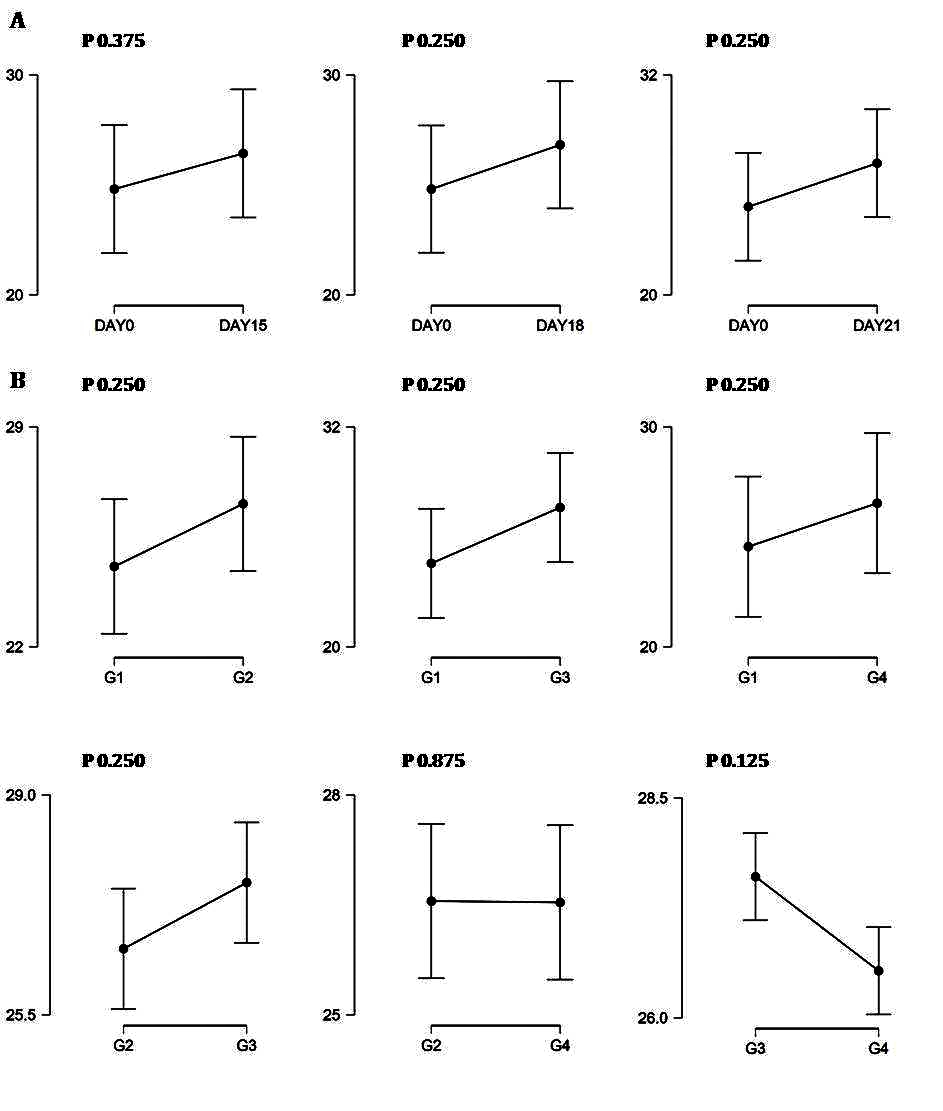


**Supplementary Figure 20:** Comparative pair-wise T-test (Wilcoxon signed-rank) outcomes for **LDL** estimations in different intervals. (A) Representation of comparative values for Day0, Day15, Day18, and Day21. (B) Comparative pair-wise T-test (Wilcoxon signed-rank) outcomes for **LDL** estimations done for G1 (control), G2, G3, and G4 groups.


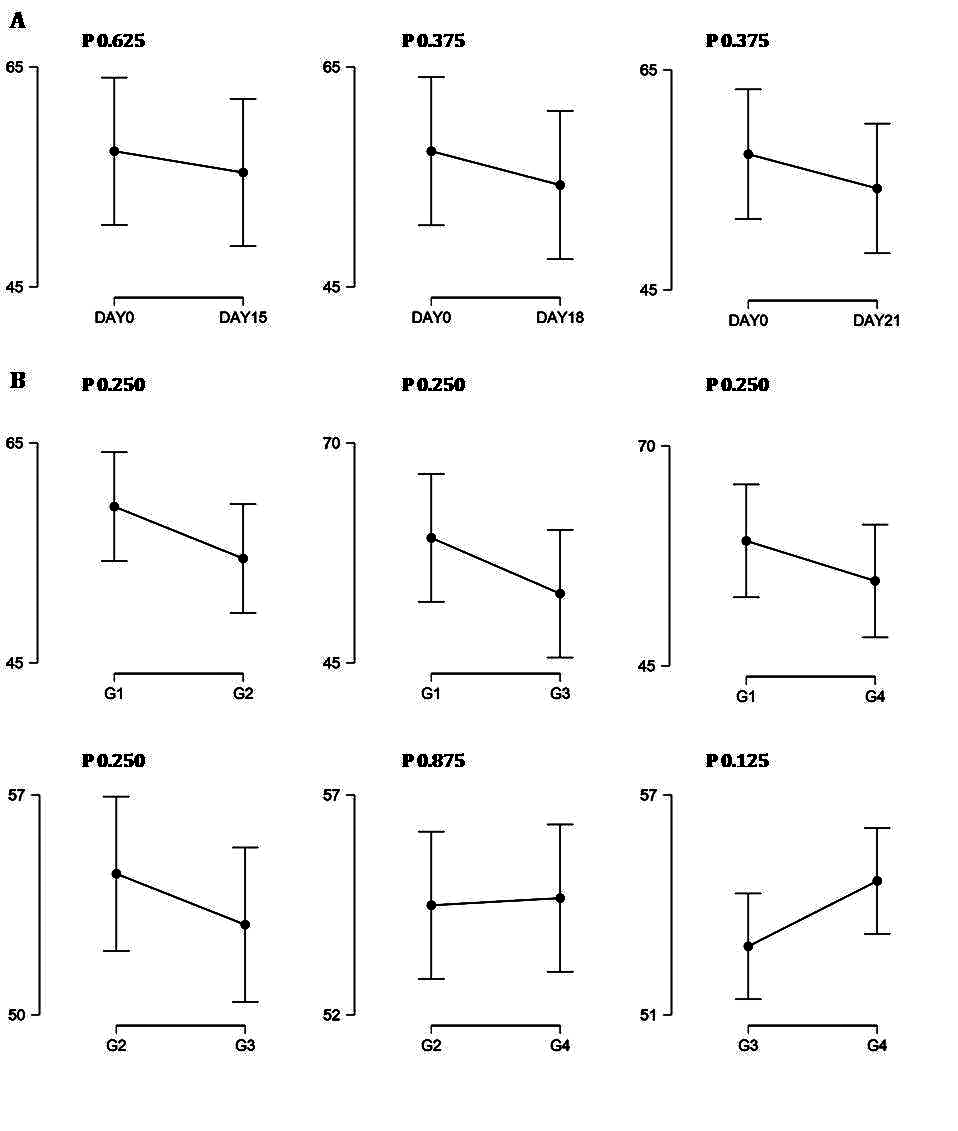


**Supplementary Figure 21:** Comparative pair-wise T-test (Wilcoxon signed-rank) outcomes for **total cholesterol** estimations in different intervals. (A) Representation of comparative values for Day0, Day15, Day18, and Day21. (B) Comparative pair-wise T-test (Wilcoxon signed-rank) outcomes for **total cholesterol** estimations done for G1 (control), G2, G3, and G4 groups.


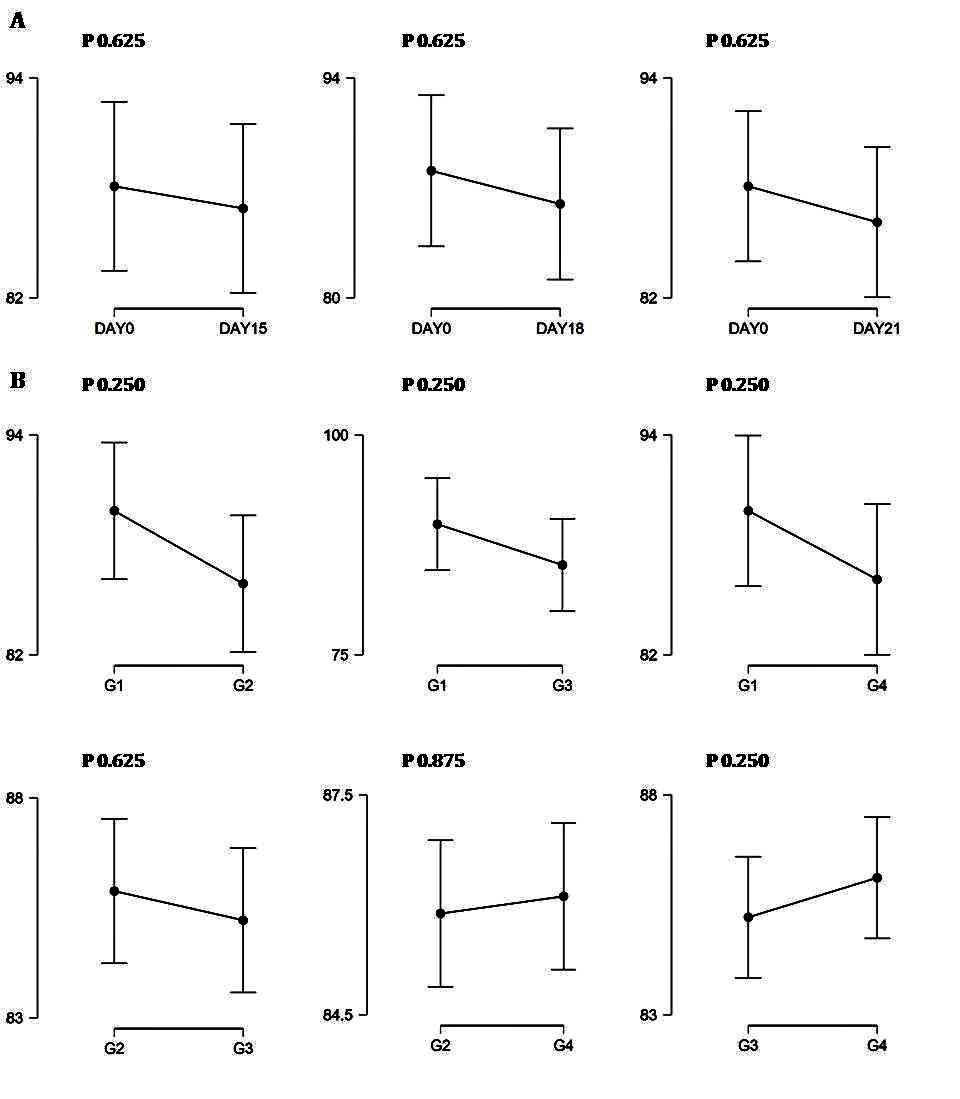


**Supplementary Figure 22:** Comparative pair-wise T-test (Wilcoxon signed-rank) outcomes for **total protein** estimations in different intervals. (A) Representation of comparative values for Day0, Day15, Day18, and Day21. (B) Comparative pair-wise T-test (Wilcoxon signed-rank) outcomes for **total protein** estimations done for G1 (control), G2, G3, and G4 groups.


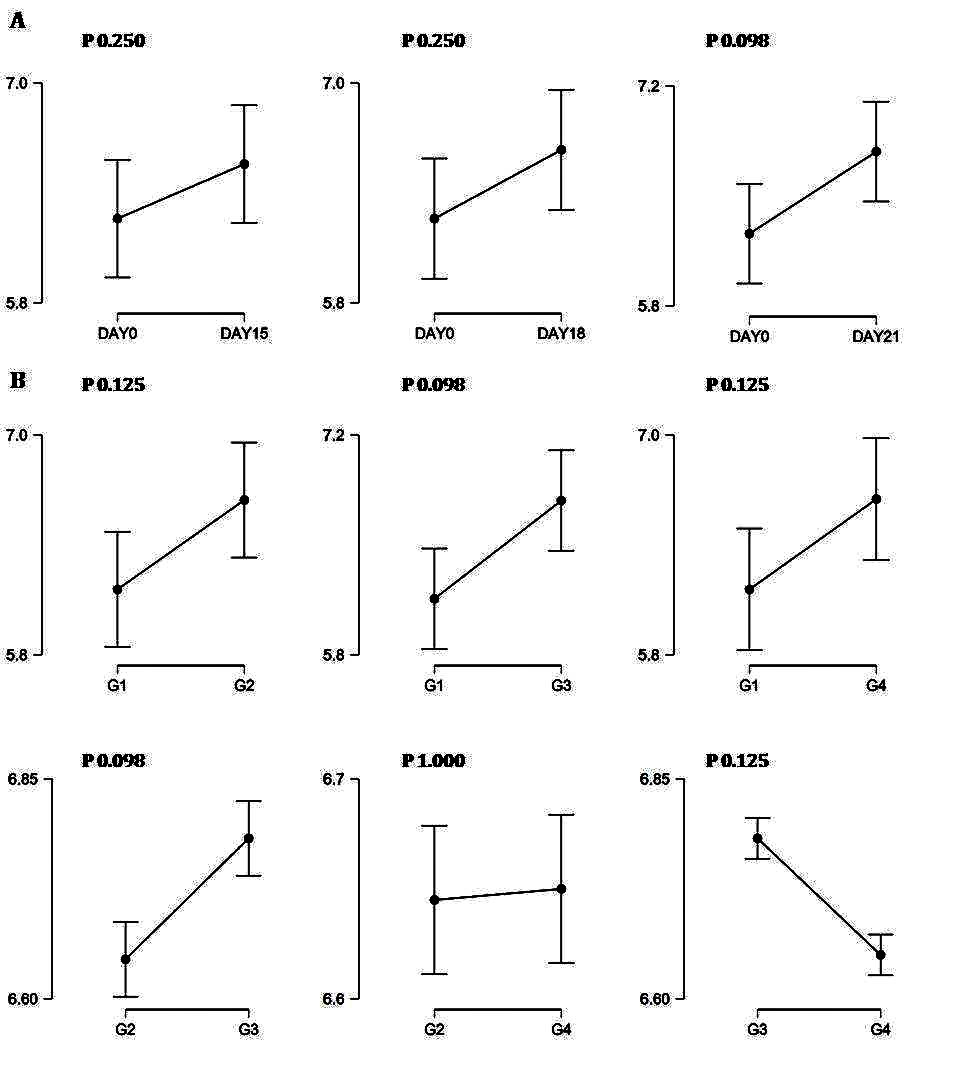


**Supplementary Figure 23:** Comparative pair-wise T-test (Wilcoxon signed-rank) outcomes for **Tryglycerides** estimations in different intervals. (A) Representation of comparative values for Day0, Day15, Day18, and Day21. (B) Comparative pair-wise T-test (Wilcoxon signed-rank) outcomes for **Tryglycerides** estimations done for G1 (control), G2, G3, and G4 groups.


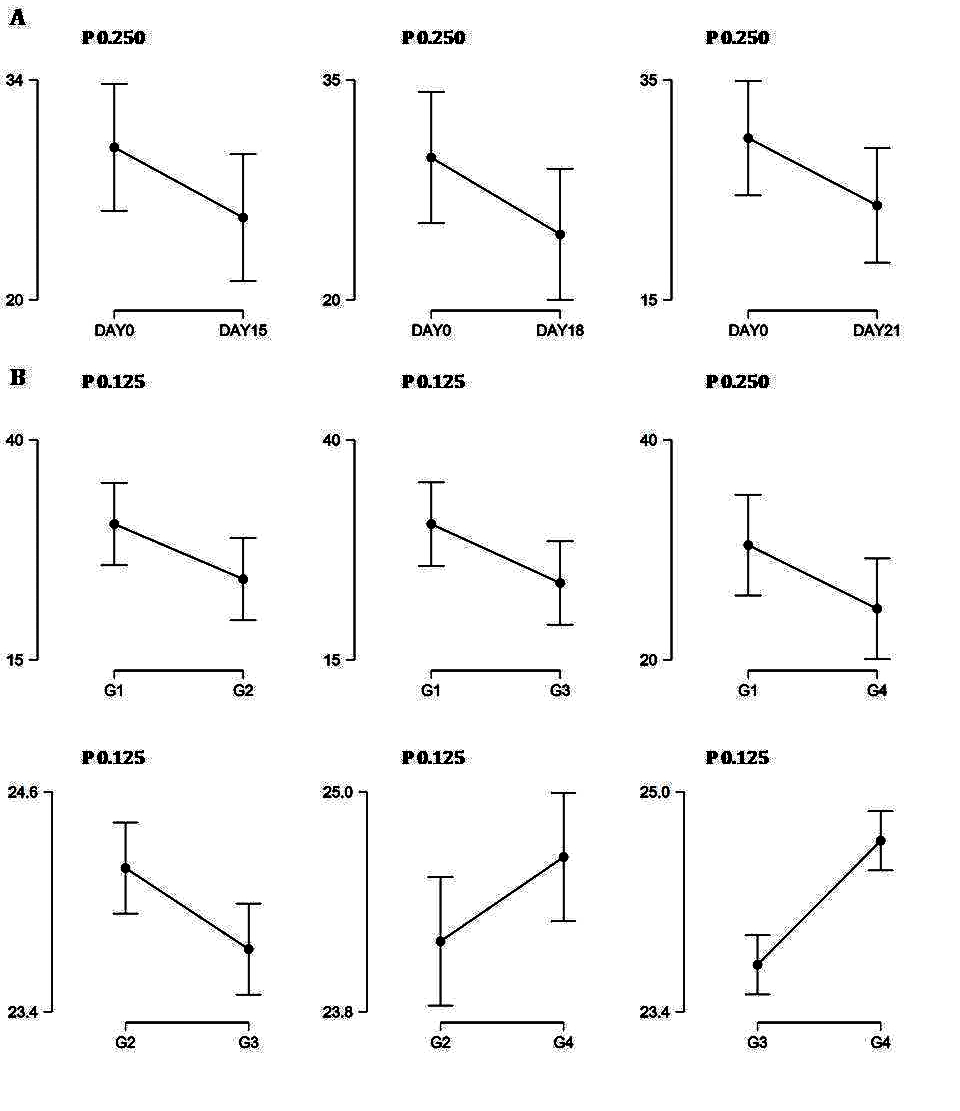


**Supplementary Figure 24:** Comparative pair-wise T-test (Wilcoxon signed-rank) outcomes for **Interleukin-6** estimations in different intervals. (A) Representation of comparative values for Day0, Day15, Day18, and Day21. (B) Comparative pair-wise T-test (Wilcoxon signed-rank) outcomes for **Interleukin-6** estimations done for G1 (control), G2, G3, and G4 groups.


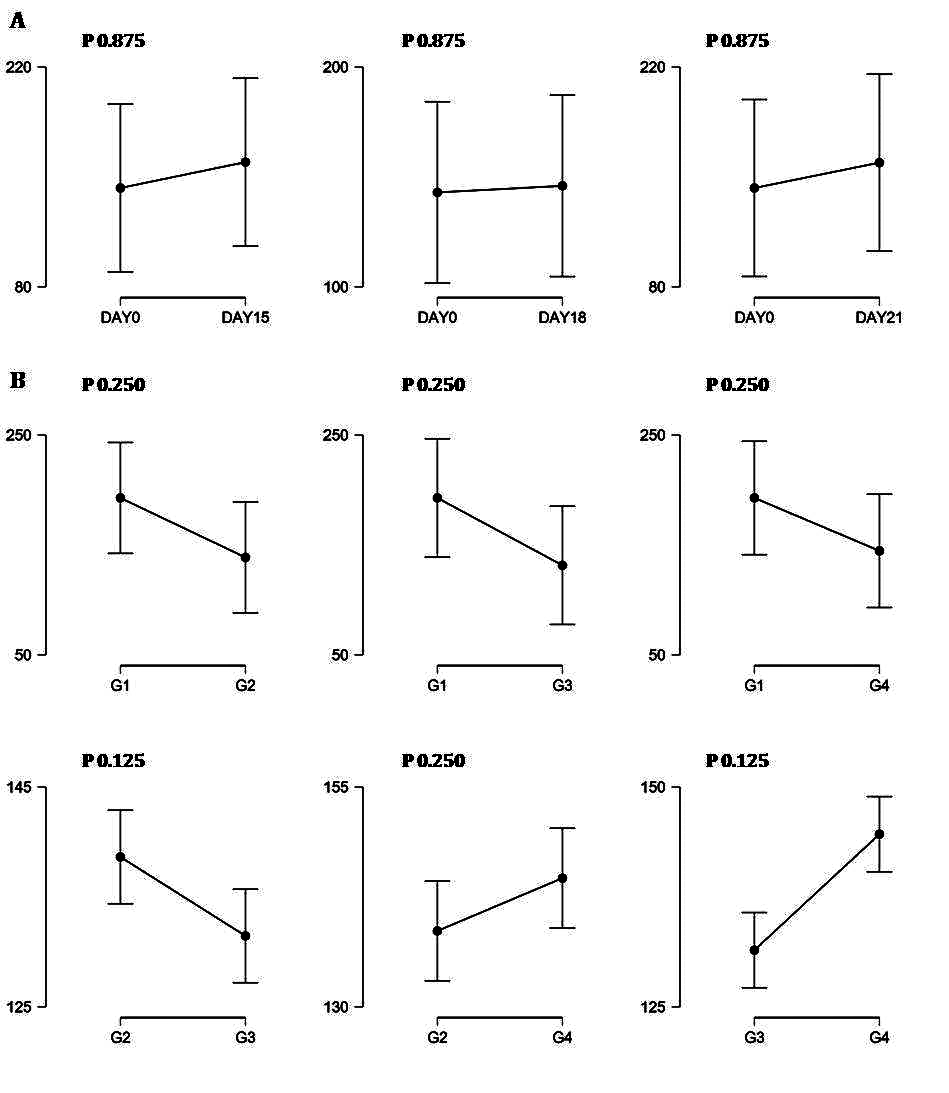

Supplement: Supplementary file 3 [file Data_Sheet_1.docx]
